# A Deficiency-Based Approach to Parametrizing Positive Equilibria of Biochemical Reaction Systems

**DOI:** 10.1007/s11538-018-00562-0

**Published:** 2018-12-31

**Authors:** Matthew D. Johnston, Stefan Müller, Casian Pantea

**Affiliations:** 1 Department of Mathematics, San José State University, One Washington Square, San Jose, CA 95192 USA; 2 Faculty of Mathematics, University of Vienna, Vienna, Austria; 3 Department of Mathematics, West Virginia University, Morgantown, WV 26506 USA

**Keywords:** Chemical reaction network, Chemical kinetics, Deficiency, Equilibrium, Algebraic variety, 92C42, 34A34

## Abstract

We present conditions which guarantee a parametrization of the set of positive equilibria of a *generalized* mass-action system. Our main results state that (1) if the underlying generalized chemical reaction network has an *effective deficiency* of zero, then the set of positive equilibria coincides with the parametrized set of complex-balanced equilibria and (2) if the network is weakly reversible and has a *kinetic deficiency* of zero, then the equilibrium set is nonempty and has a positive, typically rational, parametrization. Via the method of network translation, we apply our results to *classical* mass-action systems studied in the biochemical literature, including the EnvZ–OmpR and shuttled WNT signaling pathways. A parametrization of the set of positive equilibria of a (generalized) mass-action system is often a prerequisite for the study of multistationarity and allows an easy check for the occurrence of absolute concentration robustness, as we demonstrate for the EnvZ–OmpR pathway.

## Introduction

Networks of biochemical reactions can be represented as directed graphs where the vertices are combinations of interacting species (so-called complexes) and the edges are the reactions. Under suitable assumptions, such as spatial homogeneity and sufficient dilution, the networks follow mass-action kinetics and give rise to a system of polynomial ordinary differential equations in the species concentrations.

The mathematical study of positive equilibria of mass-action systems is important for establishing the uniqueness of equilibria in invariant regions of the state space (so-called compatibility classes) or, conversely, for establishing the capacity for multistationarity (e.g., in models of biological switches). Such analysis, however, is challenging due to the high dimensionality of the dynamical system, the significant nonlinearities, and the number of (unknown) parameters. Recent work has consequently focused on developing network-based methods for parametrizing the set of positive equilibria. Conditions for constructing monomial parametrizations (Laurent monomials) have been recently studied (Craciun et al. 2009; Pérez Millán et al. 2012; Johnston 2014; Müller and Regensburger 2014), as have conditions for constructing rational parametrizations (Thomson and Gunawardena 2009; Gross et al. 2016; Pérez Millán and Dickenstein 2016). Based on parametrizations, the uniqueness of positive equilibria has been analyzed (Conradi et al. 2008; Müller and Regensburger 2012; Müller et al. 2016; Conradi and Mincheva 2017), and regions for multistationarity (in the space of rate constants) have been identified for specific models, such as phosphorylation networks (Holstein et al. 2013; Conradi et al. 2016).

In this paper, we develop a method for explicitly constructing positive, typically rational, parametrizations of the set of positive equilibria for a broad class of biochemical reaction networks. Our approach is based on an extension of deficiency theory, the concept of generalized mass-action systems, and the method of network translation. The deficiency of a chemical reaction network was introduced in Feinberg (1972) and Horn (1972) in the context of sufficient conditions for weakly reversible networks to have complex-balanced equilibria (Horn and Jackson 1972). The notions of deficiency and complex balancing were subsequently extended to generalized mass-action systems by Müller and Regensburger (2012, 2014). Thereby, the kinetic complex determining the reaction rate was allowed to differ from the (stoichiometric) complex determining the reaction vector. Finally, the method of network translation was introduced by Johnston (2014), in order to relate a mass-action system to a generalized mass-action system that is dynamically equivalent, but has a different network structure. In particular, the translated network might be weakly reversible (even when the original network is not) and have a lower deficiency.

A generalized mass-action system for which the underlying network is weakly reversible and has deficiency zero is known to have an equilibrium set with a monomial parametrization (Müller and Regensburger 2012, 2014; Johnston 2014). In this paper, we extend this framework to construct positive parametrizations for a significantly wider class of generalized networks. To this end, we introduce a new notion of deficiency called *effective deficiency*, cf. Eq. (21), which refers to the *condensed network* of a generalized network, cf. Definition 7. Our main results state that if a weakly reversible generalized network has an effective deficiency and kinetic deficiency of zero, then the corresponding generalized mass-action system permits a positive parametrization of the set of positive equilibria. This parametrization can be computed by linear algebra techniques and does not require tools from algebraic geometry such as Gröbner bases. Via network translation, we can apply our results to a broad class of mass-action systems.

For example, consider the following two-component signaling system, which is adapted from a histidine kinase example (Conradi et al. 2016): 
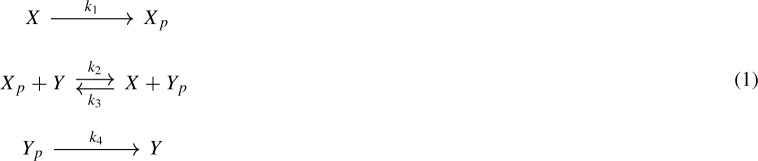
Thereby, *X* is a histidine kinase, *Y* is a response regulator, and *p* is a phosphate group. The network is not weakly reversible and has deficiency one so that the classical deficiency zero theorem does not apply. Via network translation, however, the system (1) corresponds to the following generalized mass-action system: 
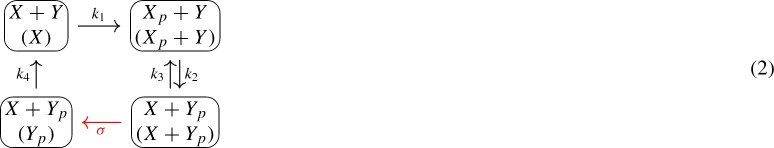
Thereby, we put a box at each vertex of the graph with the *stoichiometric complex* at the top and the *kinetic-order complex* (in brackets) at the bottom, cf. Definition 1. The red arrow corresponds to a *phantom edge*, that is, an edge which connects identical stoichiometric complexes, cf. Eq. (16). Phantom edges do not contribute to the associated system of ordinary differential equations and hence can be labeled arbitrarily. Thus, the edge label $\sigma > 0$ can be considered a free parameter.

Now, the network (2) is weakly reversible and, as it turns out, it has an *effective deficiency* of zero and a *kinetic deficiency* of zero. Our main results guarantee that the set of positive equilibria has a positive parametrization and, in fact, constructively yield the following rational parametrization:

3 $$\begin{aligned} \left\{ \begin{array}{ll} x = \displaystyle {\frac{k_4}{\sigma }},&{} \quad x_p = \displaystyle {\frac{k_1(k_3+\sigma )k_4}{k_2\sigma ^2\tau }} ,\\ y = \tau ,&{} \quad y_p = \displaystyle {\frac{k_1}{\sigma }}, \end{array}\right. \end{aligned}$$

where $\sigma , \tau > 0$. Note that the ‘rate constant’ $\sigma >0$ in the network (2) appears explicitly in the parametrization (3). Importantly, the construction of (3) via Theorem 15 depends on efficient methods from linear algebra such as generalized inverses. Our algorithm therefore represents a significant computational advantage over algebraic geometry methods such as Gröbner bases.

The paper is organized as follows: In Sect. 2, we review the relevant terminology regarding generalized chemical reaction networks and introduce several new notions, including effective and phantom edges, parametrized sets of equilibria, condensed networks, and effective deficiency. In Sect. 3, we present the crucial Lemma 13 and the main results of the paper, Theorems 14 and 15. In Sect. 4, we discuss the method of network translation, which allows us to apply the results of Sect. 3 to networks studied in the biochemical literature, such as the EnvZ–OmpR and shuttled WNT signaling pathways. In the EnvZ–OmpR example, our parametrization immediately implies the occurrence of absolute concentration robustness (ACR). In Sect. 5, we summarize our findings and present avenues for future work.

Throughout the paper, we use the following notation:

4 $$\begin{aligned}&\text{ For } v \in \mathbb {R}^{n}_{>0}, \, \ln v = (\ln v_1, \ldots , \ln v_n)^T \in \mathbb {R}^{n}. \end{aligned}$$

5 $$\begin{aligned}&\text{ For } v \in \mathbb {R}^{n}, \, e^v = (e^{v_1}, \ldots , e^{v_n})^T \in \mathbb {R}^{n}. \end{aligned}$$

6 $$\begin{aligned}&\text{ For } v \in \mathbb {R}^n_{>0} \text{ and } w \in \mathbb {R}^{n}, \, v^w = \prod _{i=1}^n v_i^{w_i} \in \mathbb {R}. \end{aligned}$$

7 $$\begin{aligned}&\text{ For } v \in \mathbb {R}^{n}_{>0} \text{ and } A \in \mathbb {R}^{n \times m}, \, v^{A} = \left( v^{a^{1}}, \ldots , v^{a^{\!m}} \right) ^T \in \mathbb {R}^m, \end{aligned}$$

8 $$\begin{aligned}&\text{ where } a^j \in \mathbb {R}^n \text{ is } \text{ the } j\text{ th } \text{ column } \text{ of } A. \nonumber \\&\text{ For } v, w \in \mathbb {R}^{n}, \, v \circ w = (v_1w_1, \ldots , v_nw_n)^T \in \mathbb {R}^{n}. \end{aligned}$$

## Mathematical Framework

We give a brief introduction to the relevant terminology regarding generalized chemical reaction networks (which include classical chemical reaction networks). In particular, we distinguish between effective and phantom edges and introduce parametrized sets of equilibria. Further, we define condensed networks and the notion of effective deficiency. Finally, we introduce the helpful concept of $V^*$-directed networks.

### Generalized Mass-Action Systems

A directed graph $G=(V,E)$ is given by a set of vertices $V = \{ 1, \ldots , m \}$ and a set of edges $E \subseteq V \times V$. We denote an edge $e=(i,j)\in E$ by $i \rightarrow j$ to emphasize that is directed from the source *i* to the target *j*. We additionally define the set of *source vertices*$V_s = \{ i \mid i \rightarrow j \in E \}$, that is, the set of vertices that appear as the source of some edge. We call the connected components of a graph *linkage classes* and the strongly connected components *strong linkage classes*. If linkage classes and strong linkage classes coincide, we call the graph *weakly reversible*.

A generalized chemical reaction network is essentially a graph with two embeddings of the vertices in $\mathbb {R}^n$. The notion was introduced by Müller and Regensburger (2012, 2014).

#### Definition 1

A *generalized chemical reaction network* (GCRN) $(G,y,{\tilde{y}})$ is given by a directed graph $G=(V,E)$ without self-loops and two maps $y{:}~V \rightarrow \mathbb {R}^n$ and ${\tilde{y}} :V_s \rightarrow \mathbb {R}^n$. Thereby, *G* is called the abstract reaction graph, and $y(i), {{\tilde{y}}}(i) \in \mathbb {R}^n$ are called the stoichiometric and kinetic-order complexes, respectively, assigned to vertex *i*.

In contrast to a classical chemical reaction network (see below), a GCRN has two complexes associated with each vertex. Thereby, the maps *y* and ${\tilde{y}}$ are not required to be injective, and the same stoichiometric or kinetic-order complex may be assigned to several vertices.

When considering examples, we represent complexes $y, {\tilde{y}} \in \mathbb {R}^n$ as formal sums of *species* (often $\{X_1, X_2, \ldots , X_n \}$). The components of the complexes correspond to the coefficients in the sums, e.g., $y = (1,0,1,0,\ldots ,0)$ is represented as $y = X_1 + X_3$.

#### Definition 2

A *generalized mass-action system* (GMAS) $(G_k,y,\tilde{y})$ is given by a GCRN $(G,y,\tilde{y})$ with $G=(V,E)$ together with edge labels $k \in \mathbb {R}^E_{>0}$, resulting in the labeled directed graph $G_k$. That is, every edge $i \rightarrow j \in E$ is labeled with a rate constant $k_{i \rightarrow j} \in \mathbb {R}_{>0}$.

The ODE system associated with a GMAS is given by

9 $$\begin{aligned} \frac{\text {d} x}{\text {d} t} = f^{G}_k(x) = \sum _{i \rightarrow j \in E} k_{i \rightarrow j} \, x^{{\tilde{y}}(i)} \, (y(j) - y(i)). \end{aligned}$$

We can rewrite the right-hand side of the ODE as

10 $$\begin{aligned} f^{G}_k(x) = Y I_E \, \text {diag}(k) (I_E^s)^T \, x^{{\tilde{Y}}} = Y A^G_k \, x^{{\tilde{Y}}} , \end{aligned}$$

where $Y, {\tilde{Y}} \in \mathbb {R}^{n \times V}$ are the matrices of stoichiometric and kinetic complexes, respectively, $I_E, I_E^s \in \mathbb {R}^{V \times E}$ are the *incidence* and *source* matrices of the graph *G*, and $A^G_k = I_E \, \text {diag}(k) (I_E^s)^T \in \mathbb {R}^{V \times V}$ is the resulting *Laplacian matrix* of the labeled directed graph $G_k$. For an example of the decomposition, see Eq. (12). The columns $y^j$ of *Y* are given by $y^j=y(j)$, and analogously for ${\tilde{Y}}$. Note that columns ${\tilde{y}}^j$ of ${\tilde{Y}}$ corresponding to non-source vertices $j \not \in V_s$ can be chosen arbitrarily since the corresponding columns $(I^s_E)^j$ of $I^s_E$ and hence the columns $(A_k^G)^j$ of $A_k^G$ are zero vectors.

Notably, the change over time (9) lies in the *stoichiometric subspace*$S = {{\,\mathrm{im}\,}}(Y I_E)$, which suggests the definition of a *stoichiometric compatibility class*$(c' + S) \cap \mathbb {R}^n_{\ge 0}$ with $c' \in \mathbb {R}^n_{\ge 0}$. The *stoichiometric deficiency* is defined as $\delta = \dim (\ker Y \cap {{\,\mathrm{im}\,}}I_E)$. Equivalently, $\delta = m - \ell - s$, where $m = |V|$ is the number of vertices, $\ell$ is the number of linkage classes of *G*, and $s = \dim S$ is the dimension of the stoichiometric subspace [e.g., see Johnston (2014)]. If $V=V_s$, that is, if every vertex is a source, we additionally define the *kinetic-order subspace*${\tilde{S}} = {{\,\mathrm{im}\,}}( {\tilde{Y}} I_E)$ and the *kinetic deficiency*${{\tilde{\delta }}} = \dim (\ker {\tilde{Y}} \cap {{\,\mathrm{im}\,}}I_E)$. Equivalently, ${\tilde{\delta }} = m - \ell - {\tilde{s}}$, where ${\tilde{s}} = \dim {\tilde{S}}$ is the dimension of the kinetic-order subspace.

#### Example 3

Consider the GCRN $(G,y,{\tilde{y}})$ with abstract graph $G = (V,E)$ given by 
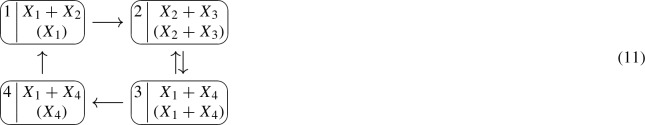
where at each vertex we display the stoichiometric complex *y* at the top and the kinetic complex ${\tilde{y}}$ (in brackets) at the bottom. That is, we have

$$\begin{aligned} y(1) = X_1+X_2, \, y(2) = X_2 + X_3, \, y(3) = y(4) = X_1 + X_4, \end{aligned}$$

and

$$\begin{aligned} {\tilde{y}}(1) = X_1, \, {\tilde{y}}(2) = X_2 + X_3, \, {\tilde{y}}(3) = X_1 + X_4, \, {\tilde{y}}(4) = X_4 . \end{aligned}$$

Note that network (2) in the introduction is essentially network (11) with specific interpretations of the species $X_1, X_2, X_3,$ and $X_4$.

This generalized network has four vertices in one linkage class and is weakly reversible. It has a two-dimensional stoichiometric subspace ($s = 2$) and a three-dimensional kinetic-order subspace (${\tilde{s}} = 3$). It follows that the stoichiometric deficiency is one ($\delta = 4-1-2 = 1$) while the kinetic deficiency is zero (${\tilde{\delta }} = 4-1-3 = 0$). The corresponding GMAS $(G_k,y)$ gives rise to the system of ODEs

12 $$\begin{aligned} \frac{\text {d} x}{\text {d} t}= & {} f^{G}_k(x) = Y A_k^G x^{{\tilde{Y}}} \nonumber \\= & {} \left[ \begin{array}{cccc} 1 &{}\quad 0 &{}\quad 1 &{}\quad 1 \\ 1 &{}\quad 1 &{}\quad 0 &{}\quad 0 \\ 0 &{}\quad 1 &{}\quad 0 &{}\quad 0 \\ 0 &{} 0 &{} 1 &{} 1 \end{array} \right] \left[ \begin{array}{cccc} -k_{1 \rightarrow 2} &{}\quad 0 &{}\quad 0 &{}\quad k_{4 \rightarrow 1} \\ k_{1 \rightarrow 2} &{}\quad -k_{2 \rightarrow 3} &{}\quad k_{3 \rightarrow 2} &{}\quad 0 \\ 0 &{}\quad k_{2 \rightarrow 3} &{}\quad -k_{3 \rightarrow 2}-k_{3 \rightarrow 4} &{}\quad 0 \\ 0 &{}\quad 0 &{}\quad k_{3 \rightarrow 4} &{}\quad -k_{4 \rightarrow 1} \end{array} \right] \left[ \begin{array}{c} x_1 \\ x_2 x_3 \\ x_1 x_4 \\ x_4 \end{array} \right] ,\nonumber \\ \end{aligned}$$

which can be expanded as

13 $$\begin{aligned} {\left\{ \begin{array}{ll} \displaystyle \frac{\text {d} x_1}{\text {d} t} =&{} -k_{1 \rightarrow 2} x_1 + k_{2 \rightarrow 3} x_2 x_3 - k_{3 \rightarrow 2} x_1 x_4 ,\\ \displaystyle \frac{\text {d} x_2}{\text {d} t} =&{} -k_{2 \rightarrow 3} x_2 x_3 + k_{3 \rightarrow 2} x_1 x_4 + k_{4 \rightarrow 1} x_4 ,\\ \displaystyle \frac{\text {d} x_3}{\text {d} t} =&{} +k_{1 \rightarrow 2} x_1 - k_{2 \rightarrow 3} x_2 x_3 + k_{3 \rightarrow 2} x_1 x_4 ,\\ \displaystyle \frac{\text {d} x_4}{\text {d} t} =&{} +k_{2 \rightarrow 3} x_2 x_3 - k_{3 \rightarrow 2} x_1 x_4 - k_{4 \rightarrow 1} x_4 . \end{array}\right. } \end{aligned}$$

### Mass-Action Systems

Classical chemical reaction networks and mass-action systems, which have been studied extensively in industrial chemistry and systems biology, can be considered as special cases of Definitions 1 and 2.

#### Definition 4

A *chemical reaction network* (CRN) is a GCRN $(G,y,{\tilde{y}})$ with $y = {\tilde{y}}$ and $y :V \mapsto \mathbb {R}^n$ being injective. A *mass-action system* (MAS) is a GMAS $(G_k,y,{\tilde{y}})$ with $y = {\tilde{y}}$ and $y :V \mapsto \mathbb {R}^n$ being injective.

Since stoichiometric and kinetic complexes agree, $y(i)={\tilde{y}}(i) \in \mathbb {R}^n$, we simply call them *complexes*. For notational convenience, we use (*G*, *y*) and $(G_k,y)$ to refer to the CRN (*G*, *y*, *y*) and the MAS $(G_k,y,y)$, respectively.

For a CRN, the stoichiometric and kinetic-order subspaces coincide (i.e., $S = {\tilde{S}}$), and the stoichiometric and kinetic deficiencies are the same (i.e., $\delta = {\tilde{\delta }}$). In fact, the *deficiency*$\delta = \dim ( \ker Y \cap {{\,\mathrm{im}\,}}I_E) = m - \ell - s$ was introduced first by Feinberg (1972) and Horn (1972) in the context of complex-balanced mass-action systems (Horn and Jackson 1972). It has been studied extensively since then (Feinberg 1979, 1987, 1995; Shinar and Feinberg 2010).

In a CRN, the map *y* is unique and vertices and complexes are in one-to-one correspondence. It is typical to write the reaction graph *G* with the complexes as vertices.

#### Example 5

Consider the CRN (*G*, *y*) given by 
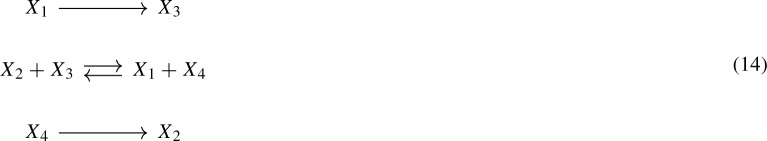


Note that network (1) in the introduction is essentially network (14) with specific interpretations of the species. The network has six vertices in three linkage classes and is not weakly reversible. It has a stoichiometric subspace of dimension two ($s=2$), and hence, its deficiency is one ($\delta = 6 - 3 - 2 = 1$).

After relabeling the rate constants, the ODE system associated with (14) is equivalent to the ODE system (13) arising from (11). Results obtained by a structural analysis of the GCRN (11) will consequently hold for the CRN given by (14). In particular, we will investigate existing methods for corresponding MASs and GMASs with equivalent dynamics in Sect. 4.1.

### Effective and Phantom Edges and Parametrized Sets of Equilibria

For a GCRN, only edges $i \rightarrow j \in E$ with $y(j) \ne y(i)$ contribute to the right-hand side of the ODE (9). In Example 3, $y(3)=y(4)$, and hence, the rate constant $k_{3 \rightarrow 4}$ does not appear in the ODEs (13), even though $3 \rightarrow 4 \in E$. Consequently, we may partition the set of edges *E* into the set of *effective edges*

15 $$\begin{aligned} E^* = \{ i \rightarrow j \in E \mid y(i) \ne y(j) \} \end{aligned}$$

and the set of *phantom edges*

16 $$\begin{aligned} E^0 = \{ i \rightarrow j \in E \mid y(i) = y(j) \}. \end{aligned}$$

Obviously, $E^* \cap E^0 = \emptyset$ and $E = E^* \cup E^0$. For a vector $k \in \mathbb {R}^E_{>0}$, we define $k^* = k_{E^*} \in \mathbb {R}^{E^*}_{>0}$ and $k^0 = k_{E^0} \in \mathbb {R}^{E^0}_{>0}$ so that, after reordering the reactions if necessary, we may write $k=(k^*,k^0)$. Further, we introduce the effective graph $G^* = (V,E^*)$.

From (9), it follows that

17 $$\begin{aligned} f^{G}_k(x)= & {} \sum _{i \rightarrow j \in E} k_{i \rightarrow j} \, x^{{\tilde{y}}(i)} \, (y(j) - y(i)) \nonumber \\= & {} \sum _{i \rightarrow j \in E^*} k_{i \rightarrow j} \, x^{\tilde{y}(i)} \, (y(j) - y(i)) = f^{G^*}_{k^*}(x) . \end{aligned}$$

That is, the GMAS $(G_k,y,{\tilde{y}})$ gives rise to the same system of ODEs as the GMAS $(G^*_k,y,{\tilde{y}})$, involving the effective graph $G^*$. In particular, the dynamics does not depend on $k^0$. From (17) and (10), it follow that

18 $$\begin{aligned} f^{G}_k(x) = f^{G}_{(k^*,k^0)}(x) = f^{G}_{(k^*,\sigma )}(x) = Y A^{G}_{(k^*,\sigma )} \, x^{{\tilde{Y}}} , \end{aligned}$$

for arbitrary $\sigma \in \mathbb {R}^{E^0}_{>0}$. That is, we may replace the rate constants $k^0$ by arbitrary parameters $\sigma$.

For a GMAS $(G_k,y,\tilde{y})$, the set of positive equilibria is given by

$$\begin{aligned} X^G_{k} := \{ x \in \mathbb {R}^n_{>0} \mid f^{G}_{k}(x) = 0 \} , \end{aligned}$$

while the set of positive *complex-balanced* equilibria (CBE) is given by

$$\begin{aligned} Z^G_k := \{ x \in \mathbb {R}^n_{>0} \mid A^G_k \, x^{{\tilde{Y}}} = 0 \} \subseteq X^G_{k} . \end{aligned}$$

Note that $X^G_k = X^{G^*}_{k^*}$, and hence, the equilibrium set $X^G_k$ depends on $k^*$, but not on $k^0$, while $Z_k$ depends on both $k^*$ and $k^0$.

Equation (18) motivates another definition. For an arbitrary parameter $\sigma \in \mathbb {R}^{E^0}_{>0}$, we consider

$$\begin{aligned} Z^G_{(k^*,\sigma )} := \{ x \in \mathbb {R}^n_{>0} \mid A^G_{(k^*,\sigma )} \, x^{{\tilde{Y}}} = 0 \} \subseteq X^G_k , \end{aligned}$$

which is the set of positive CBE of the GMAS $(G_{(k^*,\sigma )},y,\tilde{y})$. The *parametrized* set of positive CBE (PCBE) is given by

19 $$\begin{aligned} {\bar{Z}}^G_k := \bigcup _{\sigma \in \mathbb {R}^{E^0}_{>0}} Z^G_{(k^*,\sigma )} \subseteq X^G_{k} , \end{aligned}$$

thereby varying over all $\sigma \in \mathbb {R}^{E^0}_{>0}$.

For a GMAS $(G_k,y,{\tilde{y}})$, the set ${\bar{Z}}^G_k$ need not coincide with the set $X^G_{k}$. In our main results, however, we give conditions on the underlying GCRN $(G,y,{\tilde{y}})$ such that $X^G_k = {\bar{Z}}^G_k$ (Theorem 14), and also conditions under which a positive parametrization of ${\bar{Z}}^G_k$ can be constructed (Theorem 15).

#### Example 6

Recall the GCRN (11) from Example 3. The edge set *E* can be partitioned into effective edges $E^* = \{ 1 \rightarrow 2, \, 2 \rightarrow 3, \, 3 \rightarrow 2, \, 4 \rightarrow 1 \}$ and phantom edges $E^0 = \{ 3 \rightarrow 4 \}$. The equilibrium set $X_{k}$ is determined by setting the right-hand sides of the ODEs (12) to zero, whereas the set $Z_k$ of CBE is determined by the Laplacian matrix,

20 $$\begin{aligned} A_k^Gx^{{\tilde{Y}}} = \left[ \begin{array}{cccc} -k_{1 \rightarrow 2} &{}\quad 0 &{}\quad 0 &{}\quad k_{4 \rightarrow 1} \\ k_{1 \rightarrow 2} &{}\quad -k_{2 \rightarrow 3} &{}\quad k_{3 \rightarrow 2} &{}\quad 0 \\ 0 &{}\quad k_{2 \rightarrow 3} &{}\quad -k_{3 \rightarrow 2}-k_{3 \rightarrow 4} &{}\quad 0 \\ 0 &{} 0 &{} k_{3 \rightarrow 4} &{} -k_{4 \rightarrow 1} \end{array} \right] \left[ \begin{array}{c} x_1 \\ x_2 x_3 \\ x_1 x_4 \\ x_4 \end{array} \right] = \left[ \begin{array}{c} 0 \\ 0 \\ 0 \\ 0 \end{array} \right] .\nonumber \\ \end{aligned}$$

Note that these equations depend on the rate constant $k_{3 \rightarrow 4}$, even though it does not appear in the ODEs (12). By replacing $k_{3 \rightarrow 4}$ with an arbitrary parameter $\sigma$ in (20), we obtain the new set of CBE $Z_{(k^*,\sigma )}$. The set ${\bar{Z}}_{k}$ of PCBE is obtained by varying over all $\sigma \in \mathbb {R}_{>0}$. A constructive method for solving systems like (20) for the concentrations $x_i$ will be discussed in Sect. 3.2.

### Condensed Networks and Effective Deficiency

We now consider auxiliary networks with special properties. First, we introduce a network that condenses stoichiometrically identical vertices and thereby removes phantom edges.

#### Definition 7

For the GCRN $(G,y,{\tilde{y}})$, we define the *condensed* CRN $(G',y')$ given by the digraph $G' = (V', E')$, where

1. $V' = \{ [i] \mid i \in V \}$ with $[i] = \{ j \in V \mid y(j)=y(i) \}$ for $i \in V$ and
2. $$E' = \{ [i] \rightarrow [j] \mid i \rightarrow j \in E^* \}$$,

and the map $y' :V' \rightarrow \mathbb {R}^n, \, y'([i]) = y(i)$.

Note that a condensed CRN is not a GCRN and has no kinetic complexes associated with the vertices.

For the GCRN $(G,y,{\tilde{y}})$, we define the *effective* deficiency as the deficiency of its condensed CRN $(G',y')$,

21 $$\begin{aligned} \delta ' = \dim (\ker Y' \cap {{\,\mathrm{im}\,}}I_{E'}) \end{aligned}$$

with the incidence matrix $I_{E'} \in \mathbb {R}^{V' \times E'}$ and the matrix of complexes $Y' \in \mathbb {R}^{n \times V'}$, as defined after (10) in Sect. 2.1. Equivalently, $\delta ' = m' - \ell ' - s$, where $m' = |V'|$ is the number of vertices and $\ell '$ is the number of linkage classes of $G'$. Thereby, we use $S' = {{\,\mathrm{im}\,}}(Y' I_{E'}) = {{\,\mathrm{im}\,}}(Y I_E) = S$, and hence, $s' = \dim (S') = \dim (S) = s$.

Finally, we define a section $\rho :V' \rightarrow V$, assigning to each equivalence class $[i] \in V'$ a representative vertex $\rho ([i]) \in [i]$, that is, we define a set of representative vertices $V^* = \{ \rho ([i]) \mid [i] \in V' \} \subseteq V$, containing exactly one representative vertex from each equivalence class.

#### Example 8

Recall the GCRN (11) from Examples 3 and 6, in particular, that $y(3)=y(4)=X_1+X_4$. Hence, we have the equivalence classes

$$\begin{aligned}{}[1] = \{1\}, \; [2] = \{2\}, \; [3] = [4] = \{3,4\}. \end{aligned}$$

For the GCRN, we obtain its condensed CRN $(G',y')$, in particular, the graph $G'$
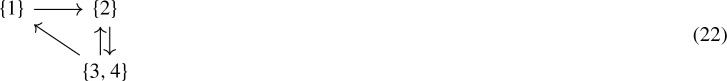
and the map $y'$ with $y'(\{1\}) = X_1$, $y'(\{2\}) = X_2 + X_3$, and $y'(\{3,4\}) = X_1 + X_4$. The deficiency of (22) is $\delta = 3-1-2=0$, that is, the effective deficiency of the GCRN (11) is $\delta ' = 0$.

### $V^*$-directed Networks

Second, we introduce a class of GCRNs which is helpful for constructing a positive parametrization of the equilibrium set.

#### Definition 9

Let $(G,y,{\tilde{y}})$ be a GCRN with $G=(V,E)$ and condensed CRN $G'=(V',E')$. Further, let $V^* \subseteq V$ be a set of representative vertices. (That is, there is a section $\rho :V' \rightarrow V$ such that $V^* = \{ \rho ([i]) \mid [i] \in V' \}$.) We say that $(G,y,{\tilde{y}})$ is $V^*$*-directed* if

$$\begin{aligned} j \rightarrow i \in E^* \quad \text {implies} \quad i \in V^*, \quad \text {that is,} \quad i = \rho ([i]), \end{aligned}$$

and

$$\begin{aligned} E^0 = \{ i \rightarrow j \mid i \in V^* , \, j \in [i]{\setminus }\{i\} \}, \end{aligned}$$

that is,

$$\begin{aligned} E^0 = \{ \rho ([i]) \rightarrow j \mid [i] \in V', \, j \in [i]{\setminus }\{\rho ([i])\} \} . \end{aligned}$$

A GCRN being $V^*$-directed guarantees that effective edges (those between equivalence classes [*i*]) enter at the representative vertex $\rho ([i]) \in V^*$, and that phantom edges (those within an equivalence class [*i*]) lead from $\rho ([i])$ to the other vertices in the class. The representative vertices $\rho ([i]) \in V^*$ may be thought of as the hubs of the representative equivalence classes through which all directed paths must travel.

#### Example 10

Recall the GCRN (11) from Examples 3, 6, and 8. Since $y(3) = y(4)$ and hence $[3]=[4]=\{3,4\}$, we have two possible sections $\rho$, that is, two possible sets of representative vertices $V^*$, namely, $V_1^* = \{1, 2, 3\}$ and $V_2^* = \{1, 2, 4\}$.

For the set $V_1^*$, effective edges enter $\{3,4\}$ at $3 = \rho (\{3,4\})$, and the phantom edge $3 \rightarrow 4$ leads from $3 = \rho (\{3,4\})$ to 4. Hence, (11) is $V_1^*$-directed. For the set $V_2^*= \{1, 2, 4\}$, the effective edge $2 \rightarrow 3$ leads to $3 \ne \rho (\{3,4\})$. Consequently, (11) is not $V_2^*$-directed.

From Example 10, the class of $V^*$-directed GCRNs may seem restrictive. The following result, however, guarantees that, for every GMAS, there is a dynamically equivalent GMAS which is $V^*$-directed, that is, the associated ODEs agree, cf. (9). This will be instrumental in applications, cf. Sect. 4.

#### Lemma 11

Let $(G,y,{\tilde{y}})$ be a GCRN with $G=(V,E)$ and representative vertex set $V^* \subseteq V$, and let $k \in \mathbb {R}_{> 0}^{E}$ be a rate vector. Then, there is a GCRN $(\hat{G},y,{\tilde{y}})$ with $\hat{G}=(V, \hat{E})$ that is $V^*$-directed and a rate vector $\hat{k} \in \mathbb {R}_{>0}^{\hat{E}}$ such that the GMASs $(G_k,y,{\tilde{y}})$ and $(\hat{G}_{\hat{k}},y,{\tilde{y}})$ are dynamically equivalent, that is, the associated ODEs agree, cf. (9).

#### Proof

First we define the set $\hat{E}^0 = \{ i \rightarrow j \mid i \in V^* , \, j \in [i]{\setminus }\{i\} \}$ and associate an arbitrary $\hat{k}_{i \rightarrow j} > 0$ to each edge $i \rightarrow j \in \hat{E}^0$. Then, we define the set $\hat{E}^* = \hat{E}^*_1 \cup \hat{E}^*_2$ as follows:

1. If $i \rightarrow j \in E^*$ and $j \in V^*$, then $i \rightarrow j \in \hat{E}^*_1$ and $\hat{k}_{i \rightarrow j} = k_{i \rightarrow j}$.
2. If $i \rightarrow j \in E^*$ and $j \not \in V^*$, then $i \rightarrow \rho ([j]) \in \hat{E}^*_2$ and $\hat{k}_{i \rightarrow \rho ([j])} = \sum _{j' \in [j]{\setminus }\{\rho ([j])\}} k_{i \rightarrow j'}.$

Now we consider the GCRN $(\hat{G}, y, y')$ with $\hat{G} = (V, \hat{E})$ and $\hat{E} = \hat{E}^0 \cup \hat{E}^*$, which is $V^*$-directed by construction. With the vector $\hat{k} \in \mathbb {R}_{> 0}^{\hat{E}}$ constructed above, we have

$$\begin{aligned} f^G_k(x)= & {} \sum _{\begin{array}{c} i \rightarrow j \in E^* \\ j \in V^* \end{array}} k_{i \rightarrow j} \, x^{{\tilde{y}}(i)} \, (y(j) - y(i)) + \sum _{\begin{array}{c} i \rightarrow j' \in E^* \\ j' \not \in V^* \end{array}} k_{i \rightarrow j'} \, x^{{\tilde{y}}(i)} \, (y(j')) - y(i)) \\= & {} \sum _{i \rightarrow j \in \hat{E}^*_1} \hat{k}_{i \rightarrow j} \, x^{{\tilde{y}}(i)} \, (y(j) - y(i)) \\&+ \sum _{i \rightarrow \rho ([j]) \in \hat{E}^*_2} \sum _{j' \in [j]{\setminus }\{\rho ([j])\}} k_{i \rightarrow j'} \, x^{{\tilde{y}}(i)} \, (y(j')) - y(i)) \\= & {} \sum _{i \rightarrow j \in \hat{E}^*_1} \hat{k}_{i \rightarrow j} \, x^{{\tilde{y}}(i)} \, (y(j) - y(i)) \\&+ \sum _{i \rightarrow \rho ([j]) \in \hat{E}^*_2} \hat{k}_{i \rightarrow \rho ([j])} \, x^{{\tilde{y}}(i)} \, (y(\rho ([j])) - y(i)) \\= & {} f^{\hat{G}}_{\hat{k}}(x) , \end{aligned}$$

where we have omitted the edge sets $E^0$ and ${\tilde{E}}^0$ according to (17). $\square$

#### Example 12

Recall from Example 10 that the GCRN (11) is not $V_2^*$-directed, where $V_2^* = \{1,2,4\}$. According to Lemma 11, however, we may replace the edge $2 \rightarrow 3$ by the edge $2 \rightarrow 4$, where $4 = \rho (\{3,4\})$. Further, we replace the phantom edge $3 \rightarrow 4$ by the phantom edge $4 \rightarrow 3$. This construction yields the following $V_2^*$-directed GCRN $(\hat{G},y, {\tilde{y}})$: 
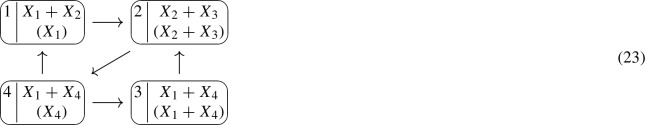
The corresponding rate vector $\hat{k} \in \mathbb {R}_{> 0}^{{\tilde{E}}}$ is $\hat{k}_{1 \rightarrow 2} = k_{1 \rightarrow 2}$, $\hat{k}_{2 \rightarrow 4} = k_{2 \rightarrow 3}$, $\hat{k}_{3 \rightarrow 2} = k_{3 \rightarrow 2}$, $\hat{k}_{4 \rightarrow 1} = k_{4 \rightarrow 1}$, and $\hat{k}_{4 \rightarrow 3} = k_{3 \rightarrow 4}$. Hence, $f_k^G = f_{\hat{k}}^{\hat{G}}$, cf. (9).

## Main Results

In Sect. 3.1, we consider GCRNs with an effective deficiency of zero ($\delta ' = 0$) and present Theorem 14, stating that the set of positive equilibria coincides with the parametrized set of complex-balanced equilibria (PCBE). In Sect. 3.2, we consider GCRNs with a kinetic deficiency of zero (${\tilde{\delta }} = 0$) and higher (${\tilde{\delta }} > 0$) and present Theorem 15, explicitly constructing the PCBE.

### Effective Deficiency

Lemma 13 below is crucial for the proof of Theorem 14. For a matrix $W \in \mathbb {R}^{n \times m}$, we write ${{\,\mathrm{cone}\,}}(W) = \{ Wx \mid x \in \mathbb {R}^m_{\ge 0} \} \subseteq \mathbb {R}^n$ for the polyhedral cone generated by the columns of *W* and ${{\,\mathrm{relint}\,}}({{\,\mathrm{cone}\,}}(W)) \subseteq \mathbb {R}^n$ for the relative interior of this cone.

#### Lemma 13

Let $(G,y,{\tilde{y}})$ be a GCRN with $G=(V,E)$ and representative vertex set $V^* \subseteq V$. In particular, let $(G,y,{\tilde{y}})$ be $V^*$-directed and have effective deficiency $\delta '=0$. Then

$$\begin{aligned} \ker Y \cap {{\,\mathrm{cone}\,}}I_{E^*} \subseteq {{\,\mathrm{cone}\,}}(-I_{E^0}). \end{aligned}$$

Moreover,

$$\begin{aligned} \ker Y \cap {{\,\mathrm{relint}\,}}({{\,\mathrm{cone}\,}}I_{E^*}) \subseteq {{\,\mathrm{relint}\,}}({{\,\mathrm{cone}\,}}(-I_{E^0})) . \end{aligned}$$

#### Proof

Let $v \in (\ker Y \cap {{\,\mathrm{cone}\,}}I_{E^*}) \subseteq \mathbb {R}^V$, that is, $v = I_{E^*} \, x = \sum _{i \rightarrow j \in E^*} x_{i \rightarrow j} \, (e_j - e_i)$ with $x \in \mathbb {R}^{E^*}_{\ge 0}$ (nonnegative weights on the effective edges $E^*$) and

$$\begin{aligned} 0 = Y v&= \sum _{i \rightarrow j \in E^*} x_{i \rightarrow j} \, (y(j) - y(i)) \\&= \sum _{[i] \rightarrow [j] \in E'} \left( \sum _{\begin{array}{c} i' \rightarrow j' \in E^*: \\ i' \in [i], j' \in [j] \end{array}} x_{i' \rightarrow j'} \right) \, (y'([j]) - y'([i])) \\&= Y' \sum _{[i] \rightarrow [j] \in E'} x'_{[i] \rightarrow [j]} \,(e_{[j]} - e_{[i]}) \\&= Y' \, I_{E'} \, x' = Y' \, v' . \end{aligned}$$

Thereby, $(G',y')$ with $G'=(V',E')$ is the corresponding condensed CRN and $v' = I_{E'} \, x' \in \mathbb {R}^{V'}$ with $x' \in \mathbb {R}^{E'}_{\ge 0}$. Clearly, $v'_{[i]} = \sum _{i' \in [i]} v_{i'}$ for $[i] \in V'$.

Now, $\delta '=\dim (\ker Y' \cap {{\,\mathrm{im}\,}}I_{E'})=0$ implies $v'=0$, that is,

$$\begin{aligned} 0 = v'_{[i]} = \sum _{i' \in [i]} v_{i'} \end{aligned}$$

for $[i] \in V'$. Using that *G* is $V^*$-directed, reconsider $v = I_{E^*} \, x \in \mathbb {R}^V$ (the fluxes arising from the effective edges $E^*$). Let $i \in V^*$, that is, $i = \rho ([i])$. For $i' \in [i]{\setminus }\{i\}$,

$$\begin{aligned} v_{i'} = - \sum _{i' \rightarrow j \in E^*} x_{i' \rightarrow j} , \end{aligned}$$

whereas

$$\begin{aligned} v_{i} = - \sum _{i' \in [i]{\setminus }\{i\}} v_{i'} . \end{aligned}$$

Now, choose ${\tilde{x}} \in \mathbb {R}^{E^0}_{\ge 0}$ (nonnegative weights on the phantom edges $E^0$) as

24 $$\begin{aligned} {\tilde{x}}_{i \rightarrow i'} = \sum _{i' \rightarrow j \in E^*} x_{i' \rightarrow j} , \end{aligned}$$

where $i' \in [i]$. Then, for $i' \in [i]{\setminus }\{i\}$,

$$\begin{aligned} v_{i'} = - {\tilde{x}}_{i \rightarrow i'} , \end{aligned}$$

whereas

$$\begin{aligned} v_{i} = - \sum _{i' \in [i]{\setminus }\{i\}} v_{i'} = \sum _{i'\in [i]{\setminus }\{i\}} {\tilde{x}}_{i \rightarrow i'} = \sum _{i \rightarrow i' \in E^0} {\tilde{x}}_{i \rightarrow i'} . \end{aligned}$$

That is, $- v = I_{E^0} \, {\tilde{x}} \in \mathbb {R}^V$ (the fluxes arising from the phantom edges $E^0$), and hence $v \in {{\,\mathrm{cone}\,}}(-I_{E^0})$.

Finally, let $v \in (\ker Y \cap {{\,\mathrm{relint}\,}}({{\,\mathrm{cone}\,}}I_{E^*}))$, that is, $v = I_{E^*} \, x$ for some $x \in \mathbb {R}^{E^*}_{>0}$. Then, $v = -I_{E^0} \, {\tilde{x}}\in {{\,\mathrm{relint}\,}}({{\,\mathrm{cone}\,}}(-I_{E^0}))$ with ${\tilde{x}} \in \mathbb {R}^{E^0}_{>0}$ by (24). $\square$

We now present the main result of this section, which gives conditions under which the equilibrium set $X_k^G$ coincides with the parametrized set of complex-balanced equilibria $\bar{Z}_k^G$.

#### Theorem 14

Let $(G,y,{\tilde{y}})$ be a GCRN with effective deficiency $\delta '=0$. Further, let $(G,y,{\tilde{y}})$ be $V^*$-directed for a set of representative vertices $V^* \subseteq V$. Then, for the GMAS $(G_k,y,{\tilde{y}})$, the set of positive equilibria agrees with the parametrized set of complex-balanced equilibria, that is, $X^G_{k} = {\bar{Z}}^G_k$.

#### Proof

Let $x \in \mathbb {R}_{>0}^n$ be a positive equilibrium, that is, $x \in X^G_k$. Using $G^*=(V,E^*)$, $G^0=(V,E^0)$, and $\sigma \in \mathbb {R}^{E^0}_{>0}$, we may write

$$\begin{aligned} A^G_{(k^*,\sigma )} \, x^{{\tilde{Y}}} = A^{G^*}_{k^{*}} \, x^{{\tilde{Y}}} + A^{G^0}_\sigma x^{{\tilde{Y}}} , \end{aligned}$$

cf. (9). Now $x \in X^G_k = X^{G^*}_{k^*}$ implies $Y A^{G^*}_{k^*} x^{{\tilde{Y}}} = 0$ and hence

$$\begin{aligned} A^{G^*}_{k^*} \, x^{{\tilde{Y}}} \in (\ker Y \cap {{\,\mathrm{relint}\,}}({{\,\mathrm{cone}\,}}I_{E^{*}})) , \end{aligned}$$

cf. (10). Since $\delta ' = 0$ and $(G,y,{\tilde{y}})$ is $V^*$-directed, we have $A^{G^*}_{k^{*}} \, x^{{\tilde{Y}}} \in {{\,\mathrm{relint}\,}}({{\,\mathrm{cone}\,}}(-I_{E^0})) ,$ by Lemma 13. That is,

$$\begin{aligned} A^{G^*}_{k^{*}} \, x^{{\tilde{Y}}} = - \sum _{i \rightarrow j \in E^0} \alpha _{i \rightarrow j} \, (e_j - e_i) \end{aligned}$$

for some $\alpha \in \mathbb {R}^{E^0}_{>0}$. On the other hand,

$$\begin{aligned} A^{G^0}_\sigma x^{{\tilde{Y}}} = \sum _{i \rightarrow j \in E^0} \sigma _{i \rightarrow j} \, x^{{\tilde{y}}(i)} \, (e_j - e_{i}). \end{aligned}$$

We choose $\sigma _{i \rightarrow j} = \alpha _{i \rightarrow j} / x^{{\tilde{y}}(i)}$ for $i \rightarrow j \in E^0$ such that $A^{G^*}_{k^*} x^{{\tilde{Y}}} = - A^{G^0}_{\sigma } x^{{\tilde{Y}}}$ and hence $A^G_{(k^*,\sigma )} \, x^{\tilde{Y}} = 0$, that is, $x \in Z^G_{(k^*,\sigma )} \subseteq {\bar{Z}}^G_k$ so $X^G_{k} \subseteq {\bar{Z}}^G_k$. Since ${\bar{Z}}^G_k \subseteq X^G_{k}$ trivially, we have $X^G_{k} = {\bar{Z}}^G_k$. $\square$

### Kinetic Deficiency

We fix the directed graph $G=(V,E)$ and omit the corresponding superscript, that is, we write $A_k^G = A_k$, $Z^G_k = Z_k$, and ${\bar{Z}}_k^G = {\bar{Z}}_k$. A subgraph *T* of $G=(V,E)$ is a directed spanning tree rooted at vertex $i \in V$ if it is a tree and, for all $j \in V$, there is a directed path from *j* to *i*.

Recall that $x \in Z_k$ is equivalent to $x^{{\tilde{Y}}} \in \ker A_{k}$. Following Johnston (2014) and Müller and Regensburger (2014), we discuss $\ker A_k$. First, we introduce the vector of *tree constants*$K \in \mathbb {R}^V_{>0}$ with entries

$$\begin{aligned} K_i = \sum _{({\mathcal {V}}, {\mathcal {E}}) \in T_i} \prod _{i' \rightarrow j' \in {\mathcal {E}}} k_{i' \rightarrow j'} , \quad i \in V , \end{aligned}$$

where $T_i$ is the set of directed spanning trees (of the respective linkage class) rooted at vertex *i*. Clearly, the tree constants *K* depend on the rate constants $k \in \mathbb {R}^E_{>0}$, that is, $K = K(k)$.

For a weakly reversible GCRN,

$$\begin{aligned} \ker A_k = {{\,\mathrm{span}\,}}\{ v^1, \ldots , v^{\ell } \} \end{aligned}$$

with nonnegative vectors $v^l \in \mathbb {R}_{\ge 0}^n$ (for $l = 1, \ldots , \ell$) having support on the respective linkage class *l*. In particular, $v^l_i = K_i$ if vertex *i* is in linkage class *l* and $v^l_i = 0$ otherwise. Now, $x^{{\tilde{Y}}} \in \ker A_k$ if and only if

$$\begin{aligned} x^{{\tilde{Y}}} = \sum _{l=1}^\ell \alpha _l \, v^l \end{aligned}$$

with $\alpha _l > 0$. For any pair of vertices *i* and *j* in the same linkage class, we have

$$\begin{aligned} \frac{x^{{\tilde{y}}(i)}}{K_{i}} = \frac{x^{{\tilde{y}}(j)}}{K_{j}}. \end{aligned}$$

Taking the logarithm gives

25 $$\begin{aligned} ({\tilde{y}}(i) - {\tilde{y}}(j))^T \ln x = \ln \left( \frac{K_{i}}{K_{j}} \right) \end{aligned}$$

where $\ln \left( v \right)$ is defined by (4).

Now we choose a spanning forest $F = (V,{\mathcal {E}})$ for $G = (V,E)$, that is, we choose spanning trees for all linkage classes. Note that *F* contains the same vertices as *G*, but not the same edges. Also note that, in the following results and applications, the choice of the spanning tree is arbitrary. Clearly, the spanning tree of linkage class *l* contains $m_l$ vertices and $m_l - 1$ edges. Hence, the spanning forest *F* contains *m* vertices and $m-\ell$ edges. We introduce the matrix $M = {\tilde{Y}} I_{\mathcal {E}} \in \mathbb {R}^{n \times {\mathcal {E}}}$ whose $m-\ell$ columns are given by ${\tilde{y}}(j) - {\tilde{y}}(i)$ for $i \rightarrow j \in {\mathcal {E}}$. Correspondingly, we define the vector $\kappa \in \mathbb {R}^{\mathcal {E}}_{>0}$ whose $m-\ell$ entries are given by $\kappa _{i \rightarrow j} = \frac{K_i}{K_j}$ for $i \rightarrow j \in {\mathcal {E}}$. As for *K*, we note that $\kappa$ depends on *k*, that is, $\kappa =\kappa (k)$. Hence, we can write the system of Eq. (25) as

26 $$\begin{aligned} M^T \ln x = \ln \kappa . \end{aligned}$$

Theorem 1 of Müller and Regensburger (2014) implies the following result.

#### Theorem 15

Let $(G,y,{\tilde{y}})$ be a GCRN that is weakly reversible, and let $(G_k,y,{\tilde{y}})$ be a GMAS. Further, let $M \in \mathbb {R}^{n \times {\mathcal {E}}}$ and $\kappa =\kappa (k)=\kappa (k^*,k^0) \in \mathbb {R}^{\mathcal {E}}_{>0}$ be defined as above, and let $H \in \mathbb {R}^{n \times {\mathcal {E}}}$ be a generalized inverse of $M^T$ (that is, $M^T H M^T = M^T$). Finally, define $B \in \mathbb {R}^{n \times (n - \tilde{s})}$ with ${{\,\mathrm{im}\,}}B = \ker M^T$ and $\ker B = \{0\}$, and $C \in \mathbb {R}^{{\mathcal {E}} \times \tilde{\delta }}$ with ${{\,\mathrm{im}\,}}C = \ker M$ and $\ker C = \{0\}$.

1. If the kinetic deficiency is zero (${\tilde{\delta }} = 0$), then ${\bar{Z}}_{k} \not = \emptyset$, in particular, ${\bar{Z}}_{k}$ has the positive parametrization
  27 $$\begin{aligned} {\bar{Z}}_{k} = \left\{ \kappa (k^*,\sigma )^{H^T} \circ \tau ^{B^T} \mid \sigma \in \mathbb {R}^{E^0}_{>0}, \quad \tau \in \mathbb {R}^{n - {\tilde{s}}}_{>0} \right\} . \end{aligned}$$
2. If the kinetic deficiency is positive (${\tilde{\delta }} > 0$) and the ${\tilde{\delta }}$ equations
  28 $$\begin{aligned} \kappa (k^*,k^0)^{C} = 1^{{\tilde{\delta }} \times 1} \end{aligned}$$
  can be solved explicitly for ${\tilde{\delta }}$ components of $k^0 \in \mathbb {R}^{E^0}_{>0}$ (in terms of $k^* \in \mathbb {R}^{E^*}_{>0}$ and the remaining components of $k^0$), that is, if there exists an explicit function $h :\mathbb {R}^{E^* \cup (E^0{\setminus }{\tilde{E}}^0)}_{>0} \rightarrow \mathbb {R}^{{\tilde{E}}^0}_{>0}$ with ${\tilde{E}}^0 \subseteq E^0$, $|\tilde{E}^0| = {\tilde{\delta }}$, and $k^0 = ({\tilde{k}}^0, \cdot ) \in \mathbb {R}^{(E^0{\setminus }{\tilde{E}}^0) \cup {\tilde{E}}^0}_{>0}$ such that, for all $k^* \in \mathbb {R}_{> 0}^{E^*}$ and ${\tilde{k}}^0 \in \mathbb {R}_{> 0}^{E^0{\setminus }{\tilde{E}}^0}$,
  $$\begin{aligned} \kappa (k^*,({\tilde{k}}^0, h(k^*,{\tilde{k}}^0)))^{C} = 1^{{\tilde{\delta }} \times 1} , \end{aligned}$$
  then ${\bar{Z}}_{k} \ne \emptyset$, and ${\bar{Z}}_{k}$ has the positive parametrization
  29 $$\begin{aligned} {\bar{Z}}_{k} = \left\{ \kappa (k^*,(\sigma , h(k^*, \sigma )))^{H^T} \circ \tau ^{B^T} \mid \sigma \in \mathbb {R}^{E^0{\setminus }{\tilde{E}}^0}_{>0}, \quad \tau \in \mathbb {R}^{n - {\tilde{s}}}_{>0} \right\} . \end{aligned}$$

Note that a matrix power is defined by (7) and ‘$\circ$’ denotes the Hadamard product, cf. (8).

Before we prove statements 1 and 2 of Theorem 15, we make two remarks.

- If the generalized inverse $H \in \mathbb {R}^{n \times {\mathcal {E}}}$ of $M^T$ has integer entries, then (27) is a rational parametrization. Common generalized inverses such as the Moore–Penrose inverse, however, rarely have this property (Ben-Israel and Greville 2003). In applications, we construct *H* by determining the matrix of elementary row operations *P* that transforms $M^T$ to reduced row echelon form. That is, we find $P \in \mathbb {R}^{{\mathcal {E}} \times {\mathcal {E}}}$ such that $PM^T \in \mathbb {R}^{{\mathcal {E}} \times n}$ is the reduced row echelon form of $M^T$. Then, we determine $Q \in \{0,1\}^{n \times {\mathcal {E}}}$ such that $Q P M^T = I$ and hence $\ln x = H \ln \kappa$ with $H = Q P \in \mathbb {R}^{n \times {\mathcal {E}}}$. That is, we perform Gaussian elimination on (26) and then set all free parameters to zero.
- As a special case of statement 2, if ${\tilde{E}}^0 = E^0$ and Eq. (28) can be solved explicitly for $k^0$ (in terms of $k^*$), that is, if there exists $h :\mathbb {R}^{E^*}_{>0} \rightarrow \mathbb {R}^{E^0}_{>0}$ such that
  $$\begin{aligned} \kappa (k^*,h(k^*))^C = 1^{{\tilde{\delta }} \times 1}, \end{aligned}$$
  then we obtain the monomial parametrization
  $$\begin{aligned} \bar{Z}_k = \left\{ \kappa (k^*,h(k^*))^{H^T} \circ \tau ^{B^T} \; | \; \tau \in \mathbb {R}_{>0}^{n - {\tilde{s}}} \right\} . \end{aligned}$$

#### Proof of statement 1

Since $(G,y,{\tilde{y}})$ is weakly reversible, $x \in Z_k$ if and only if $\ln x$ satisfies (26). Now ${{\,\mathrm{im}\,}}M = {{\,\mathrm{im}\,}}({\tilde{Y}} I_{\mathcal {E}}) = {{\,\mathrm{im}\,}}({\tilde{Y}} I_E) = {\tilde{S}}$ and hence ${{\,\mathrm{rank}\,}}M = \tilde{s}$. Since the kinetic deficiency is zero, we have $\tilde{\delta }= m - \ell - {\tilde{s}} = 0$ and hence ${\tilde{s}} = m - \ell$. That is, $M^T$ has full rank $m-\ell$ and hence $\ln \kappa \in {{\,\mathrm{im}\,}}M^T$ for any $\kappa \in \mathbb {R}_{> 0}^{m - \ell }$. Equivalently, the linear system (26) has a solution $\ln x$ for any $\kappa \in \mathbb {R}_{>0}^{\mathcal {E}}$. Following Proposition 3 of Müller and Regensburger (2014), we use the generalized inverse $H \in \mathbb {R}^{n \times {\mathcal {E}}}$ of $M^T$ and obtain

$$\begin{aligned} M^T H \ln \kappa = M^T H M^T \ln x = M^T \ln x = \ln \kappa . \end{aligned}$$

That is, $\ln x^*=H \ln \kappa$ is a solution of (26) and hence $x^* = \kappa ^{H^T} \in Z_k$. In particular, $Z_k \ne \emptyset$.

For any $$x \in Z_k$$,

$$\begin{aligned} M^T ( \ln (x) - \ln (x^*)) = 0 \end{aligned}$$

and, since $$\ker M^T = {{\,\mathrm{im}\,}}M^\perp = {\tilde{S}}^\perp $$,

$$\begin{aligned} \ln (x) - \ln (x^*) \in {\tilde{S}}^{\perp }. \end{aligned}$$

We use $B \in \mathbb {R}^{n \times (n - {\tilde{s}})}$ with ${{\,\mathrm{im}\,}}B = \tilde{S}^{\perp }$, $\ker B = \{0\}$ and obtain

$$\begin{aligned} \ln (x) - \ln ({\tilde{x}}^*) = B \alpha \end{aligned}$$

with $$\alpha \in \mathbb {R}^{n-{\tilde{s}}}$$ and

$$\begin{aligned} x = x^* \circ \tau ^{B^T} \end{aligned}$$

with $\tau = e^\alpha \in \mathbb {R}^{n-{\tilde{s}}}_{>0}$, cf. (5) and (8). Equivalently,

$$\begin{aligned} Z_k = \left\{ x^* \circ \tau ^{B^T} \mid \tau \in \mathbb {R}^{n-\tilde{s}}_{>0} \right\} = \left\{ \kappa (k^*,k^0)^{H^T} \circ \tau ^{B^T} \mid \tau \in \mathbb {R}^{n-{\tilde{s}}}_{> 0} \right\} . \end{aligned}$$

Note that the matrices *M*, *H*, and *B* do not depend on $k \in \mathbb {R}_{>0}^{E}$, whereas $\kappa = \kappa (k) = \kappa (k^*,k^0)$. Finally,

$$\begin{aligned} {\bar{Z}}_{k} = \bigcup _{\sigma \in \mathbb {R}^{E^0}_{>0}} Z_{(k^*,\sigma )} = \left\{ \kappa (k^*,\sigma )^{H^T} \circ \tau ^{B^T} \mid \sigma \in \mathbb {R}^{E^0}_{>0}, \, \tau \in \mathbb {R}^{n - {\tilde{s}}}_{>0} \right\} . \end{aligned}$$

$$\square $$

#### Proof of statement 2

If the kinetic deficiency is positive (${\tilde{\delta }} > 0$), then $M^T$ does not have full rank, and (26) does not have a solution for all right-hand sides. We use $C \in \mathbb {R}^{{\mathcal {E}} \times {\tilde{\delta }}}$ with ${{\,\mathrm{im}\,}}C = \ker M$, $\ker C = \{0\}$ and find that (26) has a solution if and only if $\ln \kappa \in {{\,\mathrm{im}\,}}M^T = \ker M^\perp = {{\,\mathrm{im}\,}}C^\perp = \ker C^T$. Equivalently, $C^T \ln \kappa = 0$, that is,

$$\begin{aligned} \kappa ^C = \kappa (k^*,k^0)^C = 1^{{\tilde{\delta }} \times 1} . \end{aligned}$$

By assumption, these ${\tilde{\delta }}$ equations can be solved explicitly for ${\tilde{\delta }}$ components of $k^0 \in \mathbb {R}^{E^0}_{>0}$ (in terms of $k^* \in \mathbb {R}^{E^*}_{>0}$ and the remaining components of $k^0$), that is, there exists an explicit function $h :\mathbb {R}^{E^* \cup (E^0{\setminus }{\tilde{E}}^0)}_{>0} \rightarrow \mathbb {R}^{{\tilde{E}}^0}_{>0}$ with ${\tilde{E}}^0 \subseteq E^0$, $|\tilde{E}^0| = {\tilde{\delta }}$, and $k^0 = ({\tilde{k}}^0, \cdot ) \in \mathbb {R}^{(E^0{\setminus }{\tilde{E}}^0) \cup {\tilde{E}}^0}_{>0}$ such that, for all $k^* \in \mathbb {R}_{> 0}^{E^*}$ and ${\tilde{k}}^0 \in \mathbb {R}_{> 0}^{E^0{\setminus }{\tilde{E}}^0}$,

$$\begin{aligned} \kappa (k^*,({\tilde{k}}^0, h(k^*,{\tilde{k}}^0))^{C} = 1^{{\tilde{\delta }} \times 1} . \end{aligned}$$

Hence, (26) has a solution for any $k^* \in \mathbb {R}^{E^*}_{>0}$ and ${\tilde{k}}^0 \in \mathbb {R}^{E^0{\setminus }\tilde{E}^0}_{>0}$, and from the proof of statement 1 we obtain the positive parametrization (29). $\square$

## Applications

The process of network translation allows to relate a classical CRN to a GCRN with potentially stronger structural properties (Johnston 2014). In Sect. 4.1, we briefly review the method, and in Sect. 4.2, we use it to apply the main results of this paper, Theorems 14 and 15, to specific mass-action systems studied in the biochemical literature.

### Translated Chemical Reaction Networks

The following definition was introduced by Johnston (2014) in order to relate a MAS to a dynamically equivalent GMAS.

#### Definition 16

Let (*G*, *y*) with $G=(V,E)$ be a CRN. A GCRN $(G^\intercal ,y^\intercal ,\tilde{y}^\intercal )$ with $G^\intercal =(V^\intercal ,E^\intercal )$ if a *translation* of (*G*, *y*) is there exists a map $g :E \rightarrow E^\intercal$ such that $g(i \rightarrow j) = i^\intercal \rightarrow j^\intercal$ with $i \rightarrow j \in E$ and $i^\intercal \rightarrow j^\intercal \in E^\intercal$ implies (i) $y^\intercal (j^\intercal )-y^\intercal (i^\intercal ) = y(j) - y(i)$ and (ii) ${\tilde{y}}^\intercal (i^\intercal ) = y(i)$.

In other words, a GCRN is a translation of a given CRN if there is a map between reactions of the two networks which (1) preserves reaction vectors and (2) relates source complexes in the CRN to kinetic complexes in the GCRN. Definition 16 is more general than Definition 6 in Johnston (2014). In that work, GCRNs were defined as by Müller and Regensburger (2012) which required $y^\intercal$ and ${\tilde{y}}^\intercal$ to be injective. Here, GCRNs are defined as by Müller and Regensburger (2014) which allows $y^\intercal$ and ${\tilde{y}}^\intercal$ to be noninjective.

#### Lemma 17

Let (*G*, *y*) be a CRN, and let $k \in \mathbb {R}^{E}_{> 0}$ be a rate vector. Further, let the GCRN $(G^\intercal , y^\intercal , \tilde{y}^\intercal )$ be a translation of (*G*, *y*), and let $k^\intercal \in \mathbb {R}_{> 0}^{E^\intercal }$ be a rate vector with $k^\intercal _{i^\intercal \rightarrow j^\intercal } = k_{i \rightarrow j}$ if $g(i \rightarrow j) = i^\intercal \rightarrow j^\intercal$. Then, the MAS $(G_k,y)$ and the GMAS $(G^\intercal _{k^\intercal }, y^\intercal , {\tilde{y}}^\intercal )$ are dynamically equivalent, that is, the associated ODEs agree, cf. (9).

#### Proof

The ODEs associated with the MAS $(G_k,y)$ and the GMAS $(G^\intercal _{k^\intercal },y^\intercal ,{\tilde{y}}^\intercal )$ are determined by (9). By Definition 16 and the construction of $k^\intercal$, we have

$$\begin{aligned} \begin{aligned} f^G_{k}(x)&= \sum _{i \rightarrow j \in E} k_{i \rightarrow j} \, x^{y(i)} \, (y(j) - y(i)) \\&= \sum _{i^\intercal \rightarrow j^\intercal \in E^\intercal } k^\intercal _{i^\intercal \rightarrow j^\intercal } \, x^{{\tilde{y}}^\intercal (i^\intercal )} \, (y^\intercal (j^\intercal ) - y^\intercal (i^\intercal )) \\&= f^{G^\intercal }_{k^\intercal }(x). \end{aligned} \end{aligned}$$

$$\square $$

Lemmas 17 and 11 provide a framework for parametrizing the set of positive equilibria of a (classical) MAS (9) by applying Theorems 14 and 15. 
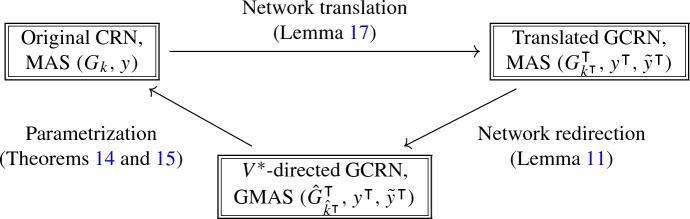


In biochemical applications, a suitable GCRN that corresponds to a given CRN may not be apparent. In particular, in order to apply Theorem 14, we want the translated network to have effective deficiency zero, and to apply Theorem 15, we want the kinetic deficiency to be as low as possible and the translated and $V^*$-directed network to be weakly reversible.

A *translation scheme* involves the addition of linear combinations of species to each side of a reaction arrow (Johnston 2014). This operation preserves reaction vectors and establishes a correspondence between source complexes in the original network and kinetic complexes in the new one. For small networks, this may suffice to create a suitably well-connected translation; however, it is extremely challenging for large networks. Computational approaches to optimal network translation have been conducted in Johnston (2015) and Tonello and Johnston (2018).

### Examples

The following examples are drawn from the biochemical literature.

#### Example 18

Recall the histidine kinase network (1) from the introduction and apply the following translation scheme: 
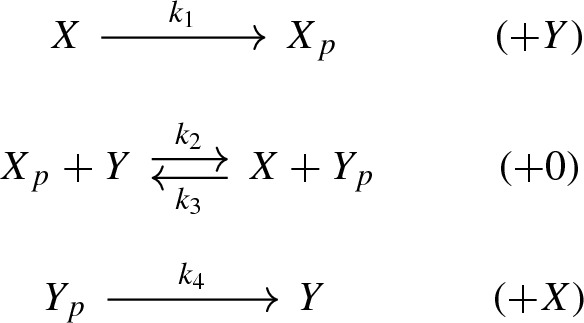
The resulting GCRN together with an additional phantom edge yields a weakly reversible GCRN, given by the (edge labeled) graph 
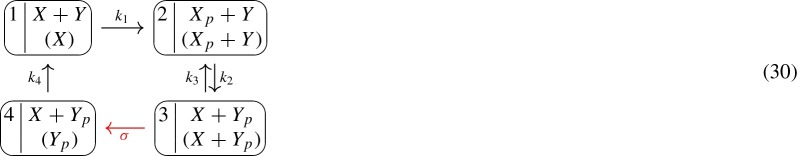
The stoichiometric complex $X+Y_p$ appears twice in (30), specifically, $[3]=[4]=\{3,4\}$, and the network is $V^*$-directed for $V^* = \{1,2,3\}$. The network has a stoichiometric deficiency of one ($\delta = 1$) and a kinetic deficiency of zero (${\tilde{\delta }} = 0)$. Its condensed network is given by the following graph: 
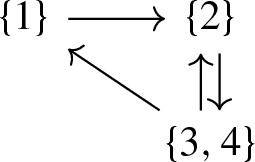
It has a deficiency of zero ($\delta ' = 0$). Theorem 14 guarantees that the equilibrium set coincides with the parametrized set of complex-balanced equilibria. Furthermore, since $\tilde{\delta }= 0$ and (30) is weakly reversible, Theorem 15 guarantees that there is a positive parametrization of the form (27).

By the construction preceding Theorem 15, we compute the matrix *M* (and further *H* and *B*). In particular, we choose a spanning forest $F = (V, {\mathcal {E}})$ for the graph (30) with edges $1 \rightarrow 2$, $1 \rightarrow 3$, and $1 \rightarrow 4$, and we compute the corresponding differences of kinetic complexes $X_p + Y - X$, $X + Y_p - X$, and $Y_p - X$:

$$\begin{aligned} M = \begin{array}{c} X \\ X_p \\ Y \\ Y_p \end{array} \left[ \begin{array}{ccc} -1 &{}\quad 0 &{}\quad -1 \\ 1 &{}\quad 0 &{}\quad 0 \\ 1 &{}\quad 0 &{}\quad 0 \\ 0 &{}\quad 1 &{}\quad 1\end{array} \right] , \qquad H = \left[ \begin{array}{ccc} 0 &{}\quad 1 &{}\quad -1 \\ 1 &{}\quad 1 &{}\quad -1 \\ 0 &{}\quad 0 &{}\quad 0 \\ 0 &{}\quad 1 &{}\quad 0 \end{array} \right] , \qquad B = \left[ \begin{array}{c} 0 \\ -1 \\ 1 \\ 0 \end{array} \right] . \end{aligned}$$

Thereby, $M^T H M^T = M^T$, that is, *H* is a generalized inverse of $M^T$, and ${{\,\mathrm{im}\,}}B = \ker M^T$.

In order to determine the parametrization (27), it remains to compute the tree constants $K=K(k^*,\sigma )$ of the graph (30) and their quotients $\kappa =\kappa (k^*,\sigma )$. We find

$$\begin{aligned} K_1 = k_2k_4\sigma , \; \; \; K_2 = k_1(k_3+\sigma )k_4, \; \; \; K_3 = k_1k_2k_4, \; \text{ and } \; K_4 = k_1k_2\sigma . \end{aligned}$$

Taking the spanning forest $F = (V, {\mathcal {E}})$ as above gives

$$\begin{aligned} \kappa _1 = \frac{K_2}{K_1} = \frac{k_1(k_3 + \sigma )}{k_2\sigma }, \quad \kappa _2 = \frac{K_3}{K_1} = \frac{k_1}{\sigma }, \quad \kappa _3 = \frac{K_4}{K_1}= \frac{k_1}{k_4}. \end{aligned}$$

As a consequence, the rational parametrization (27) amounts to

$$\begin{aligned} \left\{ \begin{array}{l} x = \kappa _2^1 \kappa _3^{-1} \cdot 1 = \frac{k_4}{\sigma } , \\ x_p = \kappa _1^1 \kappa _2^1 \kappa _3^{-1} \cdot \tau ^{-1} = \frac{k_1(k_3+\sigma )k_4}{k_2\sigma ^2\tau } , \\ y = 1 \cdot \tau ^1 = \tau , \\ y_p = \kappa _2^1 \cdot 1 = \frac{k_1}{\sigma } , \end{array}\right. \end{aligned}$$

where $$\sigma ,\tau > 0$$.

#### Example 19

Consider the following EnvZ–OmpR signaling pathway, which was first proposed by Shinar and Feinberg (2010), together with the translation scheme proposed in by Johnston (2014): 
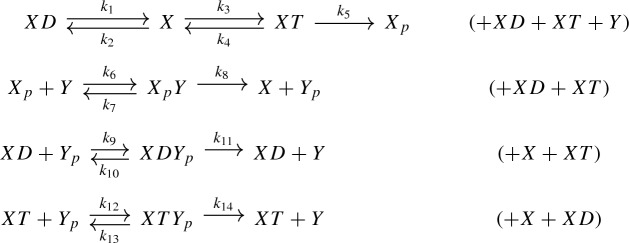
The resulting GCRN together with an additional phantom edge yields a weakly reversible GCRN, given by the (edge labeled) graph 
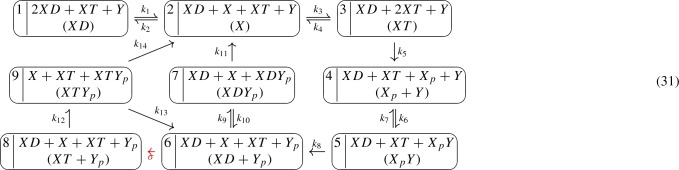
Thereby, $6 \rightarrow 8$ is the phantom edge (with label $\sigma >0$) since $y(6) = y(8) = XD + X + XT + Y_p$. The network is $V^*$-directed for $V^* = V{\setminus }\{8\}$. It can be quickly checked that the condensed graph $G'$ has deficiency zero so that (31) has an effective deficiency of zero ($\delta ' = 0$). It follows from Theorem 14 that every equilibrium point is in the parametrized set of CBE (i.e., $X_k = \bar{Z}_k$). It can also be checked that (31) has a kinetic deficiency of one (${\tilde{\delta }} = 1$). Hence, in order to apply Theorem 15 (statement 2), we need to first determine if there is $\sigma =h(k^*)$ such that $\kappa (k^*,h(k^*))^{C} = 1$.

We choose the spanning forest $F = (V, {\mathcal {E}})$ for the graph (31) consisting of the edges $1 \rightarrow i$ for $i=2, \ldots , 9$. We compute the following matrices:

$$\begin{aligned} M= & {} \begin{array}{c} XD \\ X \\ XT \\ X_p \\ Y \\ X_pY \\ Y_p \\ XDY_p \\ XTY_p \end{array} \left[ \begin{array}{cccccccc} -1 &{}\quad -1 &{}\quad -1 &{}\quad -1 &{}\quad 0 &{}\quad -1 &{}\quad -1 &{}\quad -1 \\ 1 &{}\quad 0 &{}\quad 0 &{}\quad 0 &{}\quad 0 &{}\quad 0 &{}\quad 0 &{}\quad 0\\ 0 &{}\quad 1 &{}\quad 0 &{}\quad 0 &{}\quad 0 &{}\quad 0 &{}\quad 1 &{}\quad 0\\ 0 &{}\quad 0 &{}\quad 1 &{}\quad 0 &{}\quad 0 &{}\quad 0 &{}\quad 0 &{}\quad 0\\ 0 &{}\quad 0 &{}\quad 1 &{}\quad 0 &{}\quad 0 &{}\quad 0 &{}\quad 0 &{}\quad 0\\ 0 &{}\quad 0 &{}\quad 0 &{}\quad 1 &{}\quad 0 &{}\quad 0 &{}\quad 0 &{}\quad 0\\ 0 &{}\quad 0 &{}\quad 0 &{}\quad 0 &{}\quad 1 &{}\quad 0 &{}\quad 1 &{}\quad 0\\ 0 &{}\quad 0 &{}\quad 0 &{}\quad 0 &{}\quad 0 &{}\quad 1 &{}\quad 0 &{}\quad 0\\ 0 &{}\quad 0 &{}\quad 0 &{}\quad 0 &{}\quad 0 &{}\quad 0 &{}\quad 0 &{}\quad 1 \end{array} \right] ,\\ H= & {} \left[ \begin{array}{cccccccc} 0&{}\quad 0&{}\quad 0&{}\quad 0&{}\quad 0&{}\quad 0&{}\quad 0&{}\quad -1 \\ 1&{}\quad 0&{}\quad 0&{}\quad 0&{}\quad 0&{}\quad 0&{}\quad 0&{}\quad -1 \\ 0&{}\quad 1&{}\quad 0&{}\quad 0&{}\quad 0&{}\quad 0&{}\quad 0&{}\quad -1 \\ 0&{}\quad 0&{}\quad 1&{}\quad 0&{}\quad 0&{}\quad 0&{}\quad 0&{}\quad -1 \\ 0&{}\quad 0&{}\quad 0&{}\quad 0&{}\quad 0&{}\quad 0&{}\quad 0&{}\quad 0\\ 0&{}\quad 0&{}\quad 0&{}\quad 1&{}\quad 0&{}\quad 0&{}\quad 0&{}\quad -1 \\ 0&{}\quad 0&{}\quad 0&{}\quad 0&{}\quad 1&{}\quad 0&{}\quad 0&{}\quad 0\\ 0&{}\quad 0&{}\quad 0&{}\quad 0&{}\quad 0&{}\quad 1&{}\quad 0 &{}\quad -1 \\ 0&{}0&{}0&{}0&{}0&{}0&{}0&{}0 \end{array} \right] , \; B = \left[ \begin{array}{cc} 0 &{} 1 \\ 0 &{}\quad 1 \\ 0 &{}\quad 1 \\ -1 &{}\quad 1 \\ 1 &{}\quad 0 \\ 0 &{}\quad 1 \\ 0 &{}\quad 0 \\ 0 &{}\quad 1 \\ 0 &{}\quad 1 \end{array} \right] , \; C^T = \left[ \begin{array}{c} 0 \\ -1 \\ 0 \\ 0 \\ -1 \\ 0 \\ 1 \\ 0 \end{array} \right] \end{aligned}$$

And we find the following tree constants:

$$\begin{aligned} \begin{aligned} K_{1}&= (k_4 + k_5) (((k_9 + \sigma ) k_{14} + k_9 k_{13}) k_{11} + \sigma k_{14} k_{10}) k_2 k_6 k_8 k_{12}\\ K_{2}&= (k_{4} + k_{5}) (((k_{9} + \sigma ) k_{14} + k_{9} k_{13}) k_{11} + \sigma k_{14} k_{10}) k_{1} k_{6} k_{8} k_{12}\\ K_{3}&= k_{6} (((k_{9} + \sigma ) k_{14} + k_{9} k_{13}) k_{11} + \sigma k_{14} k_{10}) k_{12} k_{1} k_{8} k_{3}\\ K_{4}&= (k_{7} + k_{8}) (((k_{9} + \sigma ) k_{14} + k_{9} k_{13}) k_{11} + \sigma k_{14} k_{10}) k_{5} k_{1} k_{3} k_{12}\\ K_{5}&= k_{5} (((k_{9} + \sigma ) k_{14} + k_{9} k_{13}) k_{11} + \sigma k_{14} k_{10}) k_{12} k_{1} k_{6} k_{3}\\ K_{6}&= (k_{10} + k_{11}) k_{12} (k_{13} + k_{14}) k_{1} k_{3} k_{5} k_{6} k_{8}\\ K_{7}&= k_{1} k_{12} k_{3} k_{5} k_{6} k_{8} k_{9} (k_{13} + k_{14})\\ K_{8}&= (k_{13} + k_{14}) k_{1} k_{3} k_{5} k_{6} k_{8} \sigma (k_{10} + k_{11})\\ K_{9}&= k_{1} k_{3} k_{5} k_{6} k_{8} \sigma (k_{10} + k_{11}) k_{12} \end{aligned} \end{aligned}$$

Constructing $\kappa =\kappa (k^*,\sigma )$ according to the spanning forest $F = (V,{\mathcal {E}})$ as above gives the ${\tilde{\delta }} = 1$ condition

$$\begin{aligned} \kappa (k^*,\sigma )^C = \left( \frac{K_{3}}{K_{1}}\right) ^{-1}\left( \frac{K_{6}}{K_{1}}\right) ^{-1} \left( \frac{K_{8}}{K_{1}}\right) = \frac{k_2(k_4+k_5)\sigma }{k_1k_3k_{12}} = 1, \end{aligned}$$

which can be solved explicitly for $\sigma$ (in terms of $k^*$),

$$\begin{aligned} \sigma = \frac{k_1k_3k_{12}}{k_2(k_4+k_5)}. \end{aligned}$$

By Theorem 15 (statement 2), we have a monomial parametrization of the form (29). In particular, we obtain:

$$\begin{aligned} \begin{aligned} XD&= \left( \frac{((k_{2} (k_{4}+k_{5}) (k_{13}+k_{14}) k_{9}+k_{1} k_{12} k_{14} k_{3}) k_{11}+k_{1} k_{3} k_{12} k_{14} k_{10}) (k_{4}+k_{5}) k_{2}}{(k_{10}+k_{11}) k_{12} k_{5} k_{3}^2 k_{1}^2 } \right) \tau _1\\ X&= \left( \frac{(((k_{2} (k_{4}+k_{5}) k_{9}+k_{1} k_{3} k_{12} k_{14}+k_{13} k_{2} k_{9} (k_{4}+k_{5})) k_{11}+k_{1} k_{3} k_{12} k_{14} k_{10}) (k_{4}+k_{5})}{ (k_{10}+k_{11}) k_{12} k_{1} k_{5} k_{3}^2} \right) \tau _1\\ XT&= \left( \frac{((k_{2} (k_{4}+k_{5}) k_{9}+k_{1} k_{3} k_{12}) k_{14}+k_{13} k_{2} k_{9} (k_{4}+k_{5})) k_{11}+k_{1} k_{3} k_{12} k_{14} k_{10}}{(k_{10}+k_{11}) k_{12} k_{1} k_{3} k_{5} } \right) \tau _1\\ X_p&= \left( \frac{(((k_{2} (k_{4}+k_{5}) k_{9}+k_{1} k_{3} k_{12}) k_{14}+k_{13} k_{2} k_{9} (k_{4}+k_{5})) k_{11}+k_{1} k_{3} k_{12} k_{14} k_{10}) (k_{7}+k_{8}) }{(k_{10}+k_{11}) k_{12} k_{3} k_{1} k_{8} k_{6}} \right) \frac{\tau _1 }{\tau _2}\\ Y&= \tau _2\\ X_pY&= \left( \frac{((k_{2} (k_{4}+k_{5}) k_{9}+k_{1} k_{3} k_{12}) k_{14}+k_{13} k_{2} k_{9} (k_{4}+k_{5})) k_{11}+k_{1} k_{3} k_{12} k_{14} k_{10}}{(k_{10}+k_{11}) k_{12} k_{3} k_{1} k_{8}} \right) \tau _1\\ Y_p&= \left( \frac{((k_{2} (k_{4}+k_{5}) k_{9}+k_{1} k_{3} k_{12}) k_{14}+k_{13} k_{2} k_{9} (k_{4}+k_{5})) k_{11}+k_{1} k_{3} k_{12} k_{14} k_{10}}{k_{5} k_{3} k_{1} (k_{13}+k_{14}) (k_{10}+k_{11})} \right) \\ XDY_p&= \left( \frac{k_{2} (k_{4}+k_{5}) (k_{13}+k_{14}) k_{9}}{(k_{10}+k_{11}) k_{1} k_{3} k_{12}} \right) \tau _1\\ XTY_p&= \tau _1 \end{aligned} \end{aligned}$$

over $\tau _1, \tau _2 \in \mathbb {R}_{>0}$. This parametrization was obtained via alternative methods by Pérez Millán et al. (2012) and Johnston (2014).

Note that the concentration of $Y_p$ does not depend upon either parameter $\tau _1$ or $\tau _2$. Hence, it takes the same value at every positive steady state. This property has been called *absolute concentration robustness* (ACR) in the literature, and the robust steady-state value of $Y_p$ has been obtained by other methods (Shinar and Feinberg 2010; Karp et al. 2012; Pérez Millán et al. 2012; Tonello and Johnston 2018).

#### Example 20

Consider the model for the Shuttled WNT signaling pathway from Gross et al. (2016), which has a deficiency of four ($\delta = 4$), taken with the following translation scheme: 
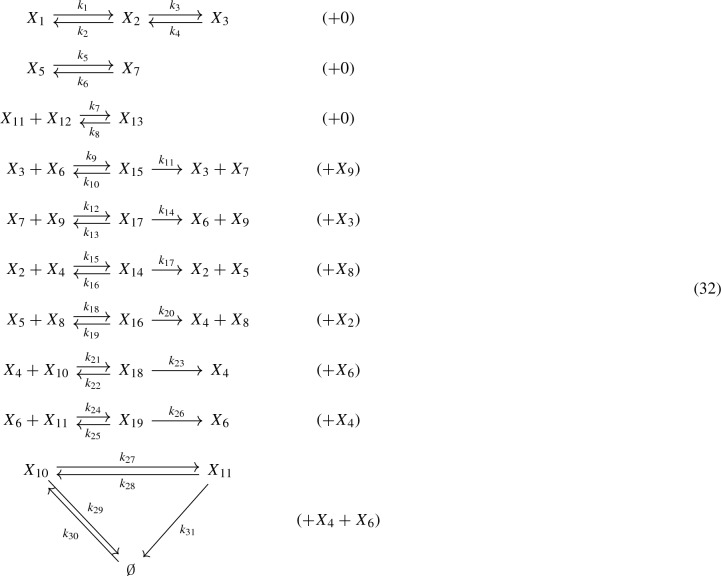
In the representation above, we have kept the indexing of the species $X_1$ through $X_{19}$ as in Gross et al. (2016), but renamed the rate constants. Via Lemmas 17 and 11, the network corresponds to a weakly reversible, $V^*$-directed GCRN:
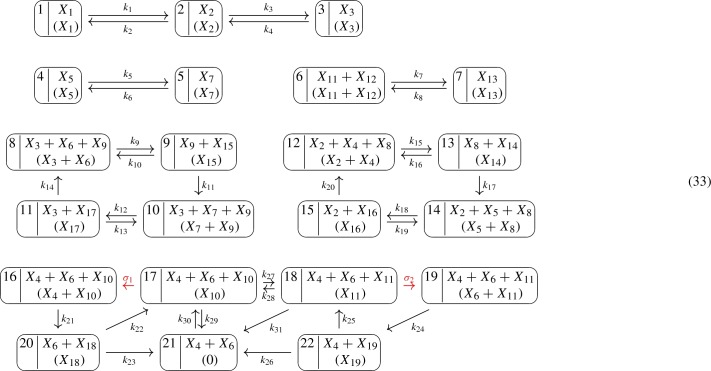
Thereby, $17 \rightarrow 16$ and $18 \rightarrow 19$ (with labels $\sigma _1> 0$ and $\sigma _2 > 0$) are phantom edges since $y(16)=y(17)=X_4+X_6+X_{10}$ and $y(18)=y(19)=X_4+X_6+X_{11}$. The network is $V^*$-directed for $V^* = V{\setminus }\{16,19\}$. It can be quickly checked that the GCRN has a stoichiometric deficiency of two ($\delta = 2$) but effective and kinetic deficiencies of zero ($\delta ' = 0$ and ${\tilde{\delta }} = 0$). By Theorems 14 and 15(statement 1), the equilibrium set can be parametrized by (27).

Explicitly, we choose the spanning forest $F = (V, {\mathcal {E}})$ for the graph (32) consisting of the edges $1 \rightarrow i$ for $i \in \{2, 3 \}$, $4 \rightarrow 5$, $6 \rightarrow 7$, $8 \rightarrow i$ for $i \in \{ 9, 10, 11\}$, $12 \rightarrow i$ for $i \in \{ 13, 14, 15 \}$, and $16 \rightarrow i$ for $i \in \{ 17, \ldots , 22 \}$. Then, we compute the corresponding matrix *M*:

$$\begin{aligned} {M = \left[ \begin{array}{cccccccccccccccc} -1&{}\quad -1&{}\quad 0&{}\quad 0&{}\quad 0&{}\quad 0&{}\quad 0&{}\quad 0&{}\quad 0&{}\quad 0&{}\quad 0&{}\quad 0&{}\quad 0&{}\quad 0&{}\quad 0&{}\quad 0\\ 1&{}\quad 0&{}\quad 0&{}\quad 0&{}\quad 0&{}\quad 0&{}\quad 0&{}\quad -1&{}\quad -1&{}\quad -1&{}\quad 0&{}\quad 0&{}\quad 0&{}\quad 0&{}\quad 0&{}\quad 0\\ 0&{}\quad 1&{}\quad 0&{}\quad 0&{}\quad -1&{}\quad -1&{}\quad -1&{}\quad 0&{}\quad 0&{}\quad 0&{}\quad 0&{}\quad 0&{}\quad 0&{}\quad 0&{}\quad 0&{}\quad 0\\ 0&{}\quad 0&{}\quad 0&{}\quad 0&{}\quad 0&{}\quad 0&{}\quad 0&{}\quad -1&{}\quad -1&{}\quad -1&{}\quad -1&{}\quad -1&{}\quad -1&{}\quad -1&{}\quad -1&{}\quad -1\\ 0&{}\quad 0&{}\quad -1&{}\quad 0&{}\quad 0&{}\quad 0&{}\quad 0&{}\quad 0&{}\quad 1&{}\quad 0&{}\quad 0&{}\quad 0&{}\quad 0&{}\quad 0&{}\quad 0&{}\quad 0\\ 0&{}\quad 0&{}\quad 0&{}\quad 0&{}\quad -1&{}\quad -1&{}\quad -1&{}\quad 0&{}\quad 0&{}\quad 0&{}\quad 0&{}\quad 0&{}\quad 1 &{}\quad 0&{}\quad 0&{}\quad 0\\ 0&{}\quad 0&{}\quad 1&{}\quad 0&{}\quad 0&{}\quad 1&{}\quad 0&{}\quad 0&{}\quad 0&{}\quad 0&{}\quad 0&{}\quad 0&{}\quad 0&{}\quad 0&{}\quad 0&{}\quad 0\\ 0&{}\quad 0&{}\quad 0&{}\quad 0&{}\quad 0&{}\quad 0&{}\quad 0&{}\quad 0&{}\quad 1&{}\quad 0&{}\quad 0 &{}\quad 0&{}\quad 0&{}\quad 0&{}\quad 0&{}\quad 0\\ 0&{}\quad 0&{}\quad 0&{}\quad 0&{}\quad 0&{}\quad 1&{}\quad 0&{}\quad 0&{}\quad 0&{}\quad 0&{}\quad 0&{}\quad 0&{}\quad 0&{}\quad 0&{}\quad 0&{}\quad 0\\ 0&{}\quad 0&{}\quad 0&{}\quad 0&{}\quad 0&{}\quad 0&{}\quad 0&{}\quad 0&{}\quad 0&{}\quad 0&{}\quad 0&{}\quad -1&{}\quad -1&{}\quad -1&{}\quad -1&{}\quad -1\\ 0&{}\quad 0&{}\quad 0&{}\quad -1&{}\quad 0&{}\quad 0&{}\quad 0&{}\quad 0&{}\quad 0&{}\quad 0&{}\quad 0&{}\quad 1&{}\quad 1&{}\quad 0&{}\quad 0&{}\quad 0\\ 0&{}\quad 0&{}\quad 0&{}\quad -1&{}\quad 0&{}\quad 0&{}\quad 0&{}\quad 0&{}\quad 0&{}\quad 0&{}\quad 0&{}\quad 0&{}\quad 0&{}\quad 0&{}\quad 0&{}\quad 0\\ 0&{}\quad 0&{}\quad 0&{}\quad 1&{}\quad 0&{} \quad 0&{}\quad 0&{} \quad 0&{}\quad 0&{}\quad 0&{}\quad 0&{}\quad 0&{}\quad 0&{}\quad 0&{}\quad 0&{}\quad 0\\ 0&{}\quad 0&{}\quad 0&{}\quad 0&{}\quad 0&{}\quad 0&{}\quad 0&{}\quad 1&{}\quad 0&{}\quad 0&{}\quad 0&{}\quad 0&{}\quad 0&{}\quad 0&{}\quad 0&{}\quad 0\\ 0&{}\quad 0&{}\quad 0&{}\quad 0&{}\quad 1&{}\quad 0&{}\quad 0&{}\quad 0&{}\quad 0&{}\quad 0&{}\quad 0&{}\quad 0&{}\quad 0&{}\quad 0&{}\quad 0&{}\quad 0\\ 0&{}\quad 0&{}\quad 0&{}\quad 0&{}\quad 0&{}\quad 0&{}\quad 0&{}\quad 0&{}\quad 0&{}\quad 1&{}\quad 0&{}\quad 0&{}\quad 0&{}\quad 0&{}\quad 0&{}\quad 0\\ 0&{}\quad 0&{}\quad 0&{}\quad 0&{}\quad 0&{}\quad 0&{}\quad 1&{}\quad 0&{}\quad 0&{}\quad 0&{}\quad 0&{}\quad 0&{}\quad 0&{}\quad 0&{}\quad 0&{}\quad 0\\ 0&{}\quad 0&{}\quad 0&{}\quad 0&{}\quad 0&{}\quad 0&{}\quad 0&{}\quad 0&{}\quad 0&{}\quad 0&{}\quad 0&{}\quad 0&{}\quad 0&{}\quad 1&{}\quad 0&{}\quad 0\\ 0&{}\quad 0&{}\quad 0&{}\quad 0&{}\quad 0&{}\quad 0&{}\quad 0&{}\quad 0&{}\quad 0&{}\quad 0&{}\quad 0&{}\quad 0&{}\quad 0&{}\quad 0&{}\quad 0&{}\quad 1 \end{array} \right] } \end{aligned}$$

We have rank $(M)=16$ and therefore nullity $(M^T)=19-16=3$. A matrix *B* with ${{\,\mathrm{im}\,}}B = \ker M^T$ and $\ker B = \{0\}$ is given by

$$\begin{aligned} B^T = \left[ \begin{array}{ccccccccccccccccccc} 1 &{}\quad 1 &{}\quad 1 &{}\quad 0 &{}\quad 1 &{}\quad 0 &{}\quad 1 &{}\quad 0 &{}\quad 0 &{}\quad 0 &{}\quad 0 &{}\quad 0 &{}\quad 0 &{}\quad 1 &{}\quad 1 &{}\quad 1 &{}\quad 1 &{}\quad 0 &{}\quad 0 \\ 0 &{}\quad 0 &{}\quad 0 &{}\quad 0 &{}\quad 0 &{}\quad 0 &{}\quad 0 &{}\quad 0 &{} \quad 0 &{}\quad 0 &{}\quad 0 &{}\quad 1&{}\quad 1 &{}\quad 0 &{}\quad 0 &{}\quad 0 &{}\quad 0 &{}\quad 0 &{}\quad 0 \\ 1 &{}\quad 1 &{}\quad 1 &{}\quad 0 &{}\quad 0 &{}\quad 0 &{}\quad 0 &{}\quad 1 &{}\quad 1 &{}\quad 0 &{}\quad 0 &{}\quad 0 &{}\quad 0 &{}\quad 1 &{}\quad 1 &{}\quad 1 &{}\quad 1 &{}\quad 0 &{}\quad 0 \end{array} \right] . \end{aligned}$$

By reducing $M^T$ to row echelon form, we obtain the following generalized inverse of $M^T$:

$$\begin{aligned} H = \left[ \begin{array}{cccccccccccccccc} 0&{}\quad -1&{}\quad 0&{}\quad 0&{}\quad 0&{}\quad -1&{}\quad 0&{}\quad 0&{}\quad 0&{}\quad 0&{}\quad 0&{}\quad 1&{}\quad -1&{}\quad 0&{}\quad 0&{}\quad 0\\ 1&{}\quad -1&{}\quad 0&{}\quad 0&{}\quad 0&{}\quad -1&{}\quad 0&{}\quad 0&{}\quad 0&{}\quad 0&{}\quad 0&{}\quad 1&{}\quad -1&{}\quad 0&{}\quad 0&{}\quad 0\\ 0&{}\quad 0&{}\quad 0&{}\quad 0&{}\quad 0&{}\quad -1&{}\quad 0&{}\quad 0&{}\quad 0&{}\quad 0&{}\quad 0&{}\quad 1&{}\quad -1&{}\quad 0&{}\quad 0&{}\quad 0\\ 0&{}\quad 0&{}\quad 0&{}\quad 0&{}\quad 0&{}\quad 0&{}\quad 0&{}\quad 0&{}\quad 0&{}\quad 0&{}\quad -1&{}\quad 0&{}\quad 0&{}\quad 0&{}\quad 0&{}\quad 0\\ 0&{}\quad 0&{}\quad -1&{}\quad 0&{}\quad 0&{}\quad -1&{}\quad 1&{}\quad 0&{}\quad 0&{}\quad 0&{}\quad 0&{}\quad 0&{}\quad 0&{}\quad 0&{}\quad 0&{}\quad 0\\ 0&{}\quad 0&{}\quad 0&{}\quad 0&{}\quad 0&{}\quad 0&{}\quad 0&{}\quad 0&{}\quad 0&{}\quad 0&{}\quad 0&{}\quad -1&{}\quad 1&{}\quad 0&{}\quad 0&{}\quad 0\\ 0&{}\quad 0&{}\quad 0&{}\quad 0&{}\quad 0&{}\quad -1&{}\quad 1&{}\quad 0&{}\quad 0&{}\quad 0&{}\quad 0&{}\quad 0&{}\quad 0&{}\quad 0&{}\quad 0&{}\quad 0\\ 1&{}\quad -1&{}\quad 1&{}\quad 0&{}\quad 0&{}\quad 0&{}\quad -1&{}\quad 0&{}\quad 0&{}\quad 1&{}\quad -1&{}\quad 1&{}\quad -1&{}\quad 0&{}\quad 0&{}\quad 0\\ 0&{}\quad 0&{}\quad 0&{}\quad 0&{}\quad 0&{}\quad 0&{}\quad 0&{}\quad 0&{}\quad 0&{}\quad 0&{}\quad 0&{}\quad 0&{}\quad 0&{}\quad 0&{}\quad 0&{}\quad 0\\ 0&{}\quad 0&{}\quad 0&{}\quad 0&{}\quad 0&{}\quad 0&{}\quad 0&{}\quad 0&{}\quad 0&{}\quad 0&{}\quad 1&{}\quad 0&{}\quad 0&{}\quad 0&{}\quad -1&{}\quad 0\\ 0&{}\quad 0&{}\quad 0&{}\quad 0&{}\quad 0&{}\quad 0&{}\quad 0&{}\quad 0&{}\quad 0&{}\quad 0&{}\quad 0&{}\quad 1&{}\quad 0&{}\quad 0&{}\quad -1&{}\quad 0\\ 0&{}\quad 0&{}\quad 0&{}\quad -1&{}\quad 0&{}\quad 0&{}\quad 0&{}\quad 0&{}\quad 0&{}\quad 0&{}\quad 0&{}\quad -1&{}\quad 0&{}\quad 0&{}\quad 1&{}\quad 0\\ 0&{}\quad 0&{}\quad 0&{}\quad 0&{}\quad 0&{}\quad 0&{}\quad 0&{}\quad 0&{}\quad 0&{}\quad 0&{}\quad 0&{}\quad 0&{}\quad 0&{}\quad 0&{}\quad 0&{}\quad 0\\ 1&{}\quad -1&{}\quad 0&{}\quad 0&{}\quad 0&{}\quad -1&{}\quad 0&{}\quad 1&{}\quad 0&{}\quad 0&{}\quad -1&{}\quad 1&{}\quad -1&{}\quad 0&{}\quad 0&{}\quad 0\\ 0&{}\quad 0&{}\quad 0&{}\quad 0&{}\quad 1&{}\quad -1&{}\quad 0&{}\quad 0&{}\quad 0&{}\quad 0&{}\quad 0&{}\quad 0&{}\quad 0&{}\quad 0&{}\quad 0&{}\quad 0\\ 1&{}\quad -1&{}\quad 0&{}\quad 0&{}\quad 0&{}\quad -1&{}\quad 0&{}\quad 0&{}\quad 1&{}\quad 0&{}\quad -1&{}\quad 1&{}\quad -1&{}\quad 0&{}\quad 0&{}\quad 0\\ 0&{}\quad 0&{}\quad 0&{}\quad 0&{}\quad 0&{}\quad 0&{}\quad 0&{}\quad 0&{}\quad 0&{}\quad 0&{}\quad 0&{}\quad 0&{}\quad 0&{}\quad 0&{}\quad 0&{}\quad 0\\ 0&{}\quad 0&{}\quad 0&{}\quad 0&{}\quad 0&{}\quad 0&{}\quad 0&{}\quad 0&{}\quad 0&{}\quad 0&{}\quad 0&{}\quad 0&{}\quad 0&{}\quad 1&{}\quad -1&{}\quad 0\\ 0&{}\quad 0&{}\quad 0&{}\quad 0&{}\quad 0&{}\quad 0&{}\quad 0&{}\quad 0&{}\quad 0&{}\quad 0&{}\quad 0&{}\quad 0&{}\quad 0&{}\quad 0&{}\quad -1&{}\quad 1 \end{array} \right] \end{aligned}$$

That is, $M^T H M^T = M^T$. From the graph (32), we obtain the tree constants $K=K(k^*,\sigma )$:

**Table:** 

$K_{1} = k_2 k_4$	$K_{8} = (k_{10}+k_{11})k_{12}k_{14}$	$K_{15} = k_{15}k_{17}k_{18}$
$K_{2} = k_1 k_4$	$K_{9} = k_9k_{12}k_{14}$	$K_{16} = (k_{22}+k_{23})k_{24}k_{30}((k_{28}+\sigma _2 +k_{31})k_{26}+k_{25}(k_{28}+k_{31}))\sigma _1$
$K_{3} = k_1 k_3$	$K_{10} = k_9k_{11}(k_{13}+k_{14})$	$K_{17} = k_{21}(k_{22}+k_{23})k_{24}k_{30}((k_{28}+\sigma _2 +k_{31})k_{26}+k_{25}(k_{28}+k_{31}))$
$K_{4} = k_6$	$K_{11} = k_9k_{11}k_{12}$	$K_{18} = k_{21}(k_{22}+k_{23})k_{24}(k_{25}+k_{26})k_{27}k_{30}$
$K_{5} = k_5$	$K_{12} = (k_{16}+k_{17})k_{18}k_{20}$	$K_{19} = k_{21}(k_{22}+k_{23})(k_{25}+k_{26})k_{27}k_{30}\sigma _2$
$K_{6} = k_8$	$K_{13} = k_{15}k_{18}k_{20}$	$K_{20} = k_{21}k_{24}k_{30}((k_{28}+\sigma _2+k_{31})k_{26}+k_{25}(k_{28}+k_{31}))\sigma _1$
$K_{7} = k_7$	$K_{14} = k_{15}k_{17}(k_{19}+k_{20})$	$K_{22} = k_{21}(k_{22}+k_{23})k_{24}k_{27}k_{30}\sigma _2$
$$K_{21}=~((((k_{28}+\sigma _2+k_{31})k_{29}+(\sigma _1+k_{27})k_{31}+\sigma _2k_{27}+\sigma _1(\sigma _2+k_{28}))k_{26}+k_{25}((k_{28}+k_{31})k_{29} +(\sigma _1+k_{27})k_{31}+\sigma _1k_{28}))k_{23} +k_{22}(((k_{28}+\sigma _2+k_{31})k_{29}+k_{27}(k_{31}+\sigma _2))k_{26}+k_{25}((k_{28}+k_{31})k_{29}+k_{31}k_{27})))k_{24}k_{21}$$		

As a result, the parametrization (27) amounts to
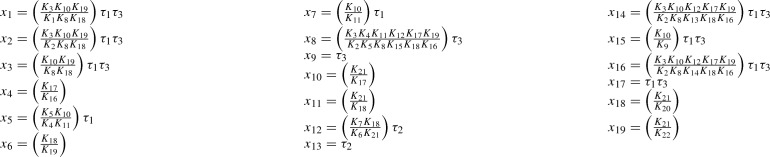


with $K=K(k^*,\sigma )$ as above and $\sigma _1, \sigma _2, \tau _1, \tau _2, \tau _3 > 0$.

## Outlook

We have presented sufficient conditions for determining whether the set of positive equilibria of a generalized mass-action system coincides with the parametrized set of complex-balanced equilibria. We have also presented sufficient conditions for guaranteeing a positive parametrization of the set of complex-balanced equilibria and for effectively constructing the parametrization. Through an extension of network translations (Johnston 2014), we have shown how the result can be immediately applied to biochemical reaction networks, including the EnvZ–OmpR signaling pathway (Shinar and Feinberg 2010) and shuttled WNT signaling pathway (Gross et al. 2016).

A number of potential avenues for further research naturally emerge from this work.

1. Recent work on generalized mass-action systems has established sign conditions sufficient for the uniqueness of equilibrium points in compatibility classes (Banaji and Pantea 2016; Müller et al. 2016). In particular, when the steady-state set is *toric* or *complex-balanced*, uniqueness and multistationarity may be established (Müller and Regensburger 2012; Pérez Millán et al. 2012). It is currently unclear, however, whether the extension to rational parametrizations in Theorem 15 might be utilized to guarantee either uniqueness or multistationarity.
2. For GCRNs with nonzero kinetic deficiency (${\tilde{\delta }} > 0$), statement 2 in Theorem 15 guarantees that, if the parameters $\sigma \in \mathbb {R}_{> 0}^{E_0}$ can be chosen to satisfy the ${\tilde{\delta }} > 0$ conditions for complex balancing, then the system has a monomial parametrization. It is currently unclear which conditions guarantee that a set of free parameters $\sigma \in \mathbb {R}_{> 0}^{E_0}$ may satisfy the ${\tilde{\delta }} > 0$ algebraic conditions on the rate parameters required for complex balancing.
3. Even for biochemical networks of moderate size, it is difficult to determine a translation scheme for constructing a GCRN corresponding to the original CRN. Computational approaches to network translation have been investigated by Johnston (2015) and Tonello and Johnston (2018). These works, however, rely on the definitions of a GCRN introduced by Müller and Regensburger (2012) and of network translation by Johnston (2014). Using the more general definitions of Müller and Regensburger (2014) would allow to extend the applicability of the computational approaches to a significantly broader class of networks.
